# Genetic Variants and Dental Caries Susceptibility: An Umbrella Review and Multilevel Meta-Analysis

**DOI:** 10.3390/genes17060724

**Published:** 2026-06-22

**Authors:** Halah Khalifa, Lina Bahanan, Ranna Yousif Johansson, Julia Naoumova, Samer Mheissen, Anna Westerlund

**Affiliations:** 1 Department of Orthodontics, Faculty of Dentistry, King Abdulaziz University, Jeddah 22254, Saudi Arabia; 2 Department of Orthodontics, Institute of Odontology, Sahlgrenska Academy, University of Gothenburg, 40530 Gothenburg, Sweden; ranna.yousif.johansson@gu.se (R.Y.J.); anna.goransson@odontologi.gu.se (A.W.); 3 Department of Dental Public Health, Faculty of Dentistry, King Abdulaziz University, Jeddah 22254, Saudi Arabia; lbahanan@kau.edu.sa; 4 Clinic of Orthodontics, Public Dental Service, Region Västra Götaland, 41390 Gothenburg, Sweden; 5 Private Practice, Anderson, SC 29621, USA

**Keywords:** caries risk, genetics, single-nucleotide polymorphism, immune response, enamel formation, taste receptors

## Abstract

**Objective**: This umbrella review aimed to evaluate the strength and consistency of evidence linking genetic variants to dental caries susceptibility. **Methods**: An umbrella review was conducted, following PRISMA 2020 guidelines. A comprehensive literature search was performed across six databases. Eligibility criteria included systematic reviews and meta-analyses of human subjects. Study selection, data extraction, and methodological quality assessment were performed systematically, with quality evaluated using the AMSTAR-2 tool. Multilevel meta-analyses were conducted to assess variant-specific and grouped genetic effects. **Results**: The search identified 29 eligible systematic reviews and meta-analyses for inclusion. The multilevel meta-analysis showed statistically significant associations for polymorphisms in *TAS2R38* rs713598 (OR = 0.26, 95% CI: 0.09–0.73) and *VDR* Cdx-2 rs11568820 (OR = 0.66, 95% CI: 0.46–0.95), both indicating lower odds of dental caries, while *MBL2* rs1800450 was associated with increased odds (OR = 1.48, 95% CI: 1.03–2.14). However, pooled effects across the main gene categories, including tooth development and mineralization, salivary composition and function, immune and inflammatory response, taste perception, and signaling, were not statistically significant. Findings were heterogeneous across studies. **Conclusions**: Current evidence on the association between genetic variants and dental caries susceptibility remains limited and inconsistent, providing insufficient support for the use of genetic markers in risk assessment or personalized prevention. The significant single-nucleotide polymorphism (SNP) associations identified in this review are hypothesis-generating and require validation in larger and more diverse populations using standardized caries definitions and gene–environment approaches.

## 1. Introduction

**Figure 1:**
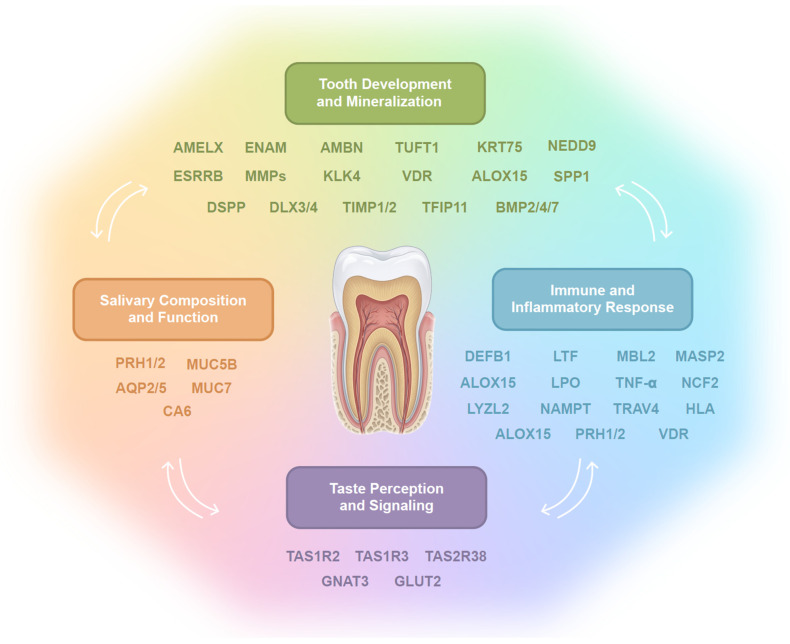
Genes associated with dental caries susceptibility are grouped by biological function. Some genes are included in more than one category due to their involvement in multiple biological pathways. Arrows indicate interactions and interrelationships among these pathways.

Variation in susceptibility to dental caries, even among individuals with comparable environmental and lifestyle risk factors, indicates an important role for genetic factors [1]. Research suggests that genetic factors account for between 49.1% and 62.7% of individual differences in caries susceptibility [2]. Consequently, genetic variants, particularly in the form of single-nucleotide polymorphisms (SNPs), have been increasingly investigated as potential risk factors for dental caries [3,4]. Genetic association studies analyze these variants using several genetic models, including allelic, dominant, and recessive, along with genotype-based comparisons (heterozygote, homozygote, and wild-type), to assess the effect of variant alleles on disease susceptibility [5,6]. Genetic variants studied in relation to dental caries have generally been grouped into four functional areas: tooth development and mineralization (TDM), salivary composition and function (SCF), immune and inflammatory responses (IMR), and taste perception and signaling (TPS). However, these categories are not mutually exclusive, and some genes may act across multiple biological pathways (Figure 1) [7,8].

The most studied genes are those involved in the formation of dental hard tissues that play an important role in regulating the development and mineralization of enamel and dentine, which serve as primary defenses against acid exposure and bacterial penetration. These genes function in a coordinated and sequential manner, regulating the process from initial protein matrix deposition to the final stages of tissue maturation, thereby influencing the strength and integrity of the tooth structure [1,8,9]. Examples of genes include amelogenin (*AMELX/AMELY*), enamelin (*ENAM*), ameloblastin (*AMBN*), tuftelin 1 (*TUFT1*), kallikrein-related peptidase 4 (*KLK4*), matrix metallopeptidases (including *MMP2*, *MMP9*, *MMP13*, and *MMP20*), and dentin sialophosphoprotein (*DSPP*) [7,10,11]. In addition, regulatory genes such as the vitamin D receptor (*VDR*) influence mineralization by regulating calcium and phosphate metabolism and maintaining mineral ion homeostasis, which is essential for tooth development [12].

Also frequently studied are genes involved in salivary composition and function, which are essential for maintaining oral health. They influence both the quality and quantity of saliva, and variations in their function can impact the protective capacity of the oral environment [8]. For example, carbonic anhydrase VI (*CA6*) influences saliva’s buffering capacity and regulates salivary pH, while aquaporin 5 (*AQP5*) regulates salivary flow [4,13,14]. Moreover, salivary glycoproteins such as mucins are encoded by the *MUC* gene family, including mucin 7 (*MUC7*) and mucin 5B (*MUC5B*) [15], and play a key role in bacterial aggregation, including that of cariogenic bacteria [4].

In addition, immune and inflammatory-related genes also play a significant role in protecting the oral cavity against microbial colonization and infection. Genes such as beta-defensin 1 (*DEFB1*) and lactoferrin (*LTF*) encode antimicrobial proteins that inhibit bacterial growth by damaging bacterial membranes and limiting the availability of essential nutrients, such as iron [4,16,17]. Additionally, mannose-binding lectin 2 (*MBL2*) and MBL-associated serine protease 2 (*MASP2*) contribute to oral immune defense through their role in the complement system, a component of innate immunity. This system enables the recognition of a broad range of pathogenic microorganisms and activation of the complement cascade [8,18]. Furthermore, *VDR* contributes to oral immune defense by regulating antimicrobial and inflammatory responses [12].

Additionally, variations in genes related to taste perception and signaling, such as taste receptor type 1 member 2 (*TAS1R2*) and taste receptor type 1 member 3 (*TAS1R3*), determine how individuals perceive sweetness, which affects their preference for sweet foods [4,19]. In parallel, the taste receptor type 2 member 38 (*TAS2R38*) gene affects sensitivity to bitter substances [4]. Individuals with specific taste receptor gene variants may be predisposed to higher sugar intake, increasing their susceptibility to dental caries [19].

Genetic factors may inherently influence individuals’ susceptibility to dental caries. Therefore, understanding the genetic basis of dental caries is essential for advancing knowledge of its etiopathogenesis, identifying high-risk groups, and facilitating effective screening and prevention strategies [20]. Moreover, a deeper understanding of these genetic factors may support the development of targeted approaches to address the complex mechanisms underlying the disease. However, existing systematic reviews report inconsistent findings and have largely examined gene categories independently, without integrating evidence across all four groups. This gap justifies the need for an umbrella review. Accordingly, this umbrella review with meta-analysis aimed to assess the association between genetic variants and the risk of dental caries in human populations, based on evidence from published systematic reviews and meta-analyses.

## 2. Materials and Methods

The umbrella review was registered in PROSPERO (registration number: CRD42024588146) and was conducted and reported in accordance with the Cochrane Handbook [21] and the Preferred Reporting Items for Systematic Reviews and Meta-Analyses (PRISMA 2020) guidelines [22]. The completed PRISMA 2020 checklist is provided in the Supplementary Material (Supplementary File S1).

### 2.1. Search Strategy and Eligibility Criteria

Published systematic reviews, with or without meta-analyses, investigating the association between any genetic variant and dental caries in human subjects of all ages, sexes, or ethnicities, and published in English, with no date restrictions, were included. Narrative or scoping reviews without systematic methods, reviews limited to non-human or laboratory studies, articles without accessible full texts, and reviews lacking outcomes of interest or sufficient detail for data extraction were excluded.

A comprehensive search strategy was developed to identify relevant systematic reviews and meta-analyses. The following electronic databases were searched: MEDLINE (through PubMed), Scopus, Web of Science, Embase, Virtual Health Library (LILACS Plus), and the Cochrane Library. The electronic database search was initially conducted in December 2024 and updated in February 2026 prior to manuscript submission to identify any newly published studies and ensure the review remained current. The search strategy was developed using Medical Subject Headings (MeSH) terms and free-text keywords related to dental caries (e.g., dental caries, dental decay, DMFT, DMFS, white spot lesions) and genetic factors (e.g., genes, genetics, polymorphism, single-nucleotide polymorphism, genome-wide association studies), combined using Boolean operators (AND, OR). The full search strategies for all databases are provided in the Supplementary Material (Supplementary File S2). A manual search of the reference lists of all included studies and relevant reviews was also conducted to identify any additional eligible publications.

### 2.2. Study Selection

Covidence (Veritas Health Innovation, Melbourne, Australia), an online platform designed to facilitate systematic reviews, was used to manage the retrieved records. After removing duplicates, the title and abstract of the studies were screened by two reviewers (HK and LB) for eligibility criteria. Similarly, the full texts of the eligible articles were assessed by two review authors (HK and LB), independently. Inter-reviewer agreement was assessed using Cohen’s kappa (κ = 0.962), indicating almost perfect agreement, and disagreements were resolved through discussion or consultation with a third reviewer (AW).

### 2.3. Data Extraction

Data extraction was carried out independently by two reviewers (HK and LB). A standardized data extraction form was used to ensure consistency and completeness across all included studies. The following data were extracted: author, year of publication, number and design of primary studies, age of participants, countries of primary studies, sample size, independent variables, outcome measurements, quality assessment tool, conclusions, and funding source. To ensure transparency and completeness, the extraction process was guided by the PRISMA checklist.

### 2.4. Quality Assessment

To evaluate the methodological quality of the included systematic reviews, we used the AMSTAR-2 tool (A Measurement Tool to Assess Systematic Reviews, version 2) [23]. The AMSTAR-2 is a validated instrument for evaluating systematic reviews, including randomized or non-randomized studies of healthcare interventions or both. The tool comprises 16 domains, including the use of a comprehensive search strategy, duplicate study selection and data extraction, risk of bias assessment, and the meta-analysis methodologies (Supplementary File S3). According to AMSTAR-2, each systematic review was rated high, moderate, low, or critically low. Each review was assessed independently by two reviewers (HK and LB), and any disagreements were resolved by a third reviewer (JN).

### 2.5. Data Synthesis

Data collection and synthesis followed the instructions in the Cochrane Handbook [21]. Comparable data from primary studies were extracted from the included systematic reviews while avoiding duplication. Primary study data were extracted for each SNP from the systematic review that included the largest number of studies in the meta-analysis for that SNP. If more than one systematic review included different primary studies, data were extracted from each SR only once. Including overlapped findings from the same primary study in the meta-analysis was avoided (Supplementary Table S1). As a result, neither primary-study data nor corresponding effect estimates were duplicated in the meta-analysis. Effect estimates were extracted as reported in the included reviews and reanalyzed in the multilevel meta-analysis. Different genetic models were retained as reported in the original reviews. No additional harmonization of allele coding was performed beyond the information provided in the included reviews. The odds ratio was the measure of interest for the association between gene polymorphisms and dental caries. Due to the dependency of data within genes (multiple polymorphisms) and within countries or studies, a multilevel meta-analysis [24] was performed. Clustering at the gene-level and country or study-level was modeled as random effects, while polymorphism-based genetic models and genotype were specified as moderators. The hierarchical structure comprised effect sizes nested within SNPs, SNPs nested within genes, and genes nested within studies (or within the country of the study team). The variance components were estimated using the restricted maximum-likelihood (REML) method. The allelic genotype was set as the reference group, and all other genotype comparisons were evaluated against this reference group to estimate the odds ratio for each examined genotype relative to the reference genotype. The SNPs were tested against the null value of 1 to determine whether they were associated with an increased or decreased risk of dental caries. All statistical tests were conducted by the author (SM) using the metafor package [25] in R software (version 4.5.0, Vienna, Austria). All tests were two-sided at a 0.05 significance level. If combining the results was not possible using multilevel meta-analysis due to a low number of studies or insufficient information, the Jadad algorithm [26] was applied to select and report findings from the most appropriate systematic review (Supplementary Table S2). Publication bias was assessed visually using funnel plots, as the application of formal statistical tests was not feasible in the context of the multilevel meta-analysis.

## 3. Results

### 3.1. Study Selection

**Figure 2:**
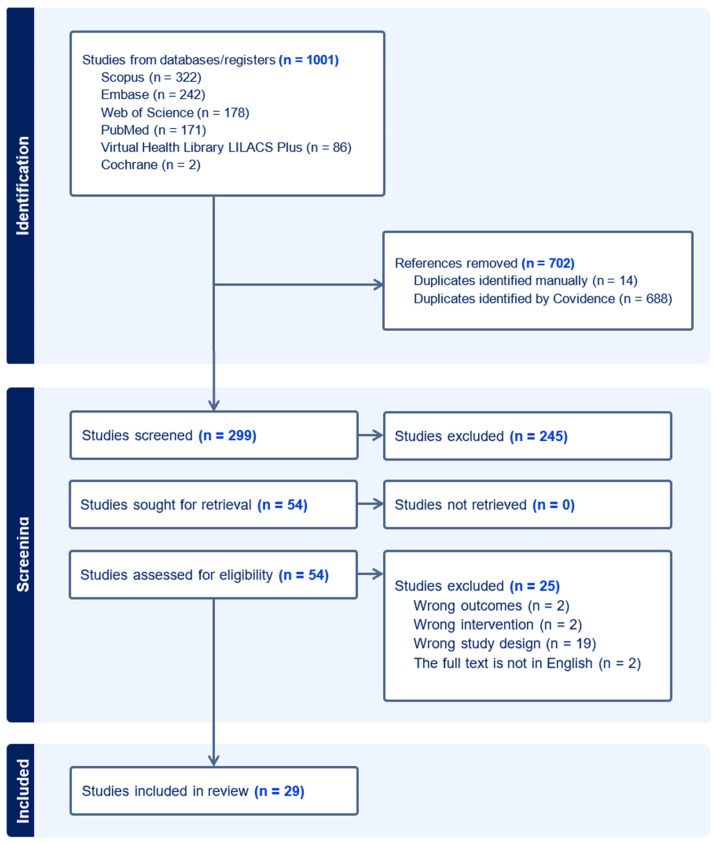
PRISMA flow diagram.

A total of 1001 articles were identified across six databases. Following the removal of 702 duplicates, 299 records were screened by title and abstract. Of these, 54 full-text articles were assessed for eligibility. Twenty-five studies were excluded based on study design (n = 19), intervention (n = 2), outcome (n = 2), or language restrictions (n = 2; Supplementary Table S3). Consequently, 29 systematic reviews [7,10,11,13,20,27,28,29,30,31,32,33,34,35,36,37,38,39,40,41,42,43,44,45,46,47,48,49,50] were included in this umbrella review. The study selection process is illustrated in the PRISMA flow diagram (Figure 2).

### 3.2. Characteristics of Included Studies

The included reviews investigated associations between genetic variants and dental caries susceptibility across diverse populations and age groups (Supplementary Table S4 provides a list of genes, SNPs, and their classification). Most reviews included observational study designs, predominantly case–control designs, with fewer cross-sectional and cohort studies, and only one review focused on genome-wide association studies [46]. The number of included studies per review ranged from 4 to 51 studies. The age of participants across the included reviews was broad, spanning from early childhood (approximately 20 months) to older adulthood (up to 93 years), although some studies did not report age ranges [33,36,37]. Sample sizes ranged from fewer than 10 to more than 50,000 participants. Geographically, the included reviews covered diverse populations from multiple regions (Table 1). A condensed summary of the included reviews is presented in Table 1, while detailed study characteristics are provided in Supplementary Table S5. 

### 3.3. Quality Assessment

**Figure 3:**
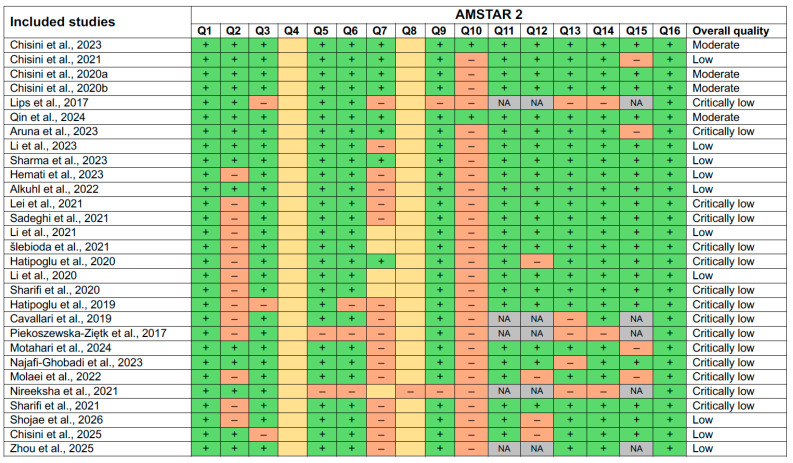
Methodological quality assessment of included reviews using AMSTAR 2 tool [7,10,11,13,20,27,28,29,30,31,32,33,34,35,36,37,38,39,40,41,42,43,44,45,46,47,48,49,50]. Color coding: green (+) = Yes; yellow (blank) = Partial yes; red (−) = No; gray (NA) = Not applicable.

Four reviews were rated as moderate in quality [47,48,49,50], ten as low [10,11,39,40,41,42,43,44,45,46], and fifteen were rated as critically low [7,13,20,27,28,29,30,31,32,33,34,35,36,37,38]. The most common methodological limitations included a lack of protocol registration, inadequate literature search strategies, and incomplete reporting of excluded studies. Additionally, most reviews did not adequately describe included studies and often failed to report funding sources (Figure 3).

### 3.4. Data Synthesis

#### 3.4.1. Genes Related to Tooth Development and Mineralization

**Figure 4:**
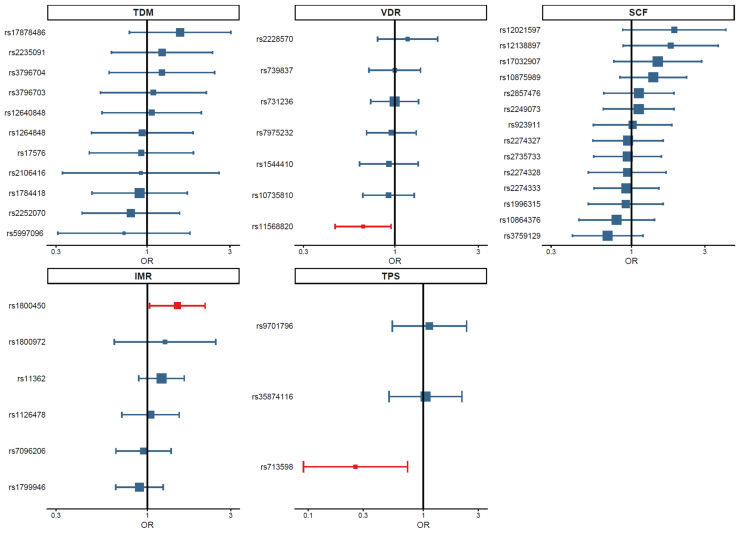
Forest plot showing the association between single-nucleotide polymorphisms (SNPs) and dental caries susceptibility grouped into five categories: tooth development and mineralization (TDM), vitamin D receptor (*VDR*), salivary composition and function (SCF), immune and inflammatory responses (IMR) and taste perception and signaling (TPS) genes. Odds ratios (ORs) and 95% confidence intervals are presented for each SNP. The vertical line indicates no association (OR = 1). Statistically significant results are highlighted in red.

Findings from the included systematic reviews indicated that the most frequently studied genes were *ENAM*, *AMELX*, *MMP13*, *AMBN*, *TUFT1*, *KLK4*, and *MMP20* (Table 1). The unadjusted pooled effect showed a statistically significant protective effect of tooth development genes on dental caries (OR = 0.94, 95% CI 0.91–0.97; Q = 498.02, *p* < 0.001). However, this association disappeared after accounting for clustering and moderators in the multilevel model (OR = 1.01, 95% CI 0.86–1.17). Variability was observed in the association between tooth development gene polymorphisms and the risk of dental caries; however, none of these results reached a statistically significant level. Furthermore, less variability was observed in the association with dental caries across genetic models and genotypes within this gene group, with a trend of decreased odds of dental caries (Table 2 and Figure 4).

A separate analysis was conducted for the *VDR* gene. Across the included systematic reviews, multiple polymorphisms of the *VDR* gene, including FokI, BsmI, TaqI, ApaI, BglI, and Cdx-2, were investigated (Table 1). The pooled odds ratio for the *VDR* gene was 0.96, with no statistically significant association with dental caries. All included polymorphisms FokI, BsmI, TaqI, and ApaI showed a non-significant association with dental caries. However, the *VDR* polymorphism (Cdx-2 rs11568820) was associated with 34% lower odds of caries, and this was statistically significant (*p* = 0.03). The heterozygous genotype was associated with a 25% increase in the odds of caries, which was marginally significant. The over-dominant model showed a significant 17% increase in odds compared with the allelic model (Table 2 and Figure 4).

#### 3.4.2. Genes Related to Salivary Composition and Function

Findings from the included systematic reviews indicated that *CA6*, *AQP5*, *MUC7*, and *MUC5B* were the most frequently studied genes (Table 1). In the quantitative synthesis, *AQP2* polymorphisms (rs12138897 and rs12021597) were associated with 80% and 90% increased odds of dental caries, respectively; however, these associations were not statistically significant. Furthermore, all included salivary genes, *MUC5B*, *CA6*, and *AQP5*, showed varying degrees of association with dental caries; however, none reached a statistically significant level. Likewise, the homozygous genotype showed increased odds of dental caries for this gene, but this was not statistically significant (Table 2 and Figure 4).

#### 3.4.3. Genes Related to Immune and Inflammatory Responses

Across the included systematic reviews, *DEFB1*, *LTF*, *MBL2*, and *MASP2* were the most frequently investigated immune and inflammatory-related genes (Table 1). The meta-analysis showed that the *MBL2* polymorphism (rs1800450) was associated with a 48% increase in the odds of dental caries (OR = 1.48, 95% CI: 1.03–2.14), and this association was statistically significant (*p* = 0.04). Increased odds of caries were also observed for two *DEFB1* polymorphisms (rs11362 and rs1800972); however, these associations were not statistically significant. In contrast, homozygous genotype and recessive model were associated with lower odds of caries, although these associations were not statistically significant (Table 2 and Figure 4).

#### 3.4.4. Genes Related to Taste Perception and Signaling

The included systematic reviews reported that *TAS2R38* and *TAS1R2* were among the most investigated genes (Table 1). The meta-analysis found that *TAS2R38* polymorphism (rs713598) was associated with 74% lower odds of dental caries, indicating that this polymorphism has a statistically significant protective effect. A trend was noted in the genotypes within this group, but this was not statistically significant (Table 2 and Figure 4).

#### 3.4.5. Publication Bias

**Figure 5:**
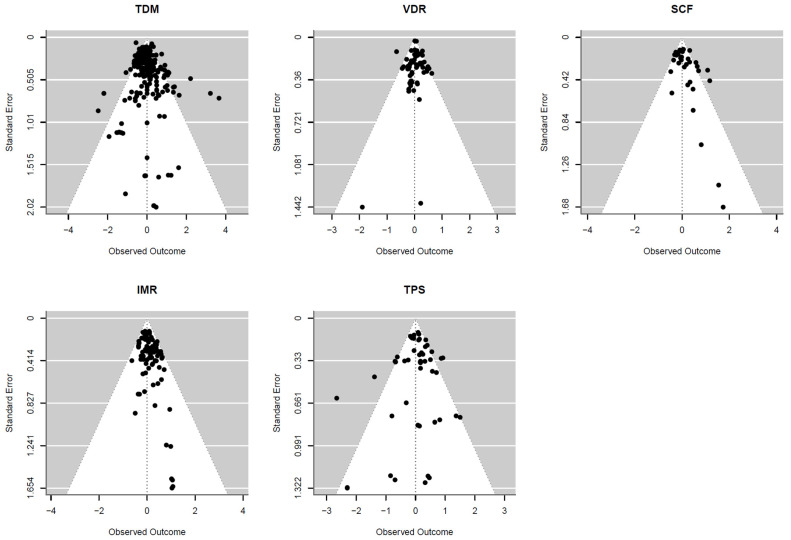
Funnel plots evaluating publication bias for studies examining the association between single-nucleotide polymorphisms (SNPs) and dental caries across five categories: tooth development and mineralization (TDM), vitamin D receptor (*VDR*), salivary composition and function (SCF), immune and inflammatory responses (IMR) and taste perception and signaling (TPS) genes. Each dot represents an individual study; Symmetry of the plots suggests low risk of publication bias, whereas asymmetry may indicate potential bias or small-study effects.

Considering the structure of multilevel meta-analysis, funnel plots showed some degree of asymmetry. This may reflect a small study effect or publication bias. However, the dependency of effect sizes and the small number of studies available for several SNPs and gene categories limited the reliability of the publication bias assessment. Therefore, the findings should be interpreted with caution (Figure 5).

## 4. Discussion

This umbrella review synthesized evidence from multiple systematic reviews and meta-analyses to investigate the association between genetic variants and dental caries risk. The findings highlight the complexity of the underlying mechanisms of dental caries and support the view that dental caries is a multifactorial condition, in which genetic susceptibility interacts with biological, environmental, and behavioral factors rather than being driven by single genetic determinants [1,51]. Among IMR genes, the *MBL2* polymorphism (rs1800450) was associated with increased odds of dental caries. This finding is consistent with its biological role, as *MBL2* is involved in innate immune defense and pathogen recognition [4,8]. Variations in this gene may impair host defense against cariogenic bacteria, thereby increasing susceptibility to dental caries. However, the available evidence remains inconsistent, with some studies reporting no significant association [52,53] and others identifying associations with different *MBL2* polymorphisms [54]. Similarly, the *VDR* polymorphism (Cdx-2 rs11568820) was associated with reduced odds of dental caries. However, findings for *VDR* remain inconsistent across studies, with some reporting associations with other *VDR* polymorphisms [29,47].

The *TAS2R38* polymorphism (rs713598) also showed a protective effect. This association may be explained by the role of *TAS2R38* in bitter taste perception, which can influence dietary behavior. Variations in this gene can affect preferences for sweet foods, which may increase sugar intake and potentially lead to a higher risk of dental caries. In this context, genetic effects may act indirectly through behavior rather than directly on tooth structure or saliva [19,55,56]. Overall, these findings highlight the complex interplay between genetic factors and dietary habits in determining caries risk [1].

An important observation in this review is the discrepancy between associations identified at the variant-level and those observed at the gene-group level. While some individual polymorphisms (e.g., *TAS2R38*, *MBL2*, and *VDR* Cdx-2) showed statistically significant associations, these effects were not consistently observed in the pooled estimates at the gene-group level. This suggests that genetic influences on dental caries are generally modest and may vary depending on the level of analysis.

Several widely studied genes across different pathways, including TDM genes such as *AMELX* and *ENAM*, IMR genes such as *DEFB1*, and SCF genes such as *CA6* and *AQP5*, did not show statistically significant associations in the present analysis, despite their established biological relevance. These findings contrast with previous studies reporting associations between these genes and dental caries susceptibility [7,20]. An important factor to consider is the high level of variability across studies. Differences in populations, age groups, sample sizes, study designs, caries assessment methods, and analytical approaches limited the comparability of findings. For instance, some associations were observed only in specific ethnic groups [29,30,41,47] or differed between primary [11,27,43,44] and permanent dentition [28,29,47]. These findings support the view of dental caries as a complex, multifactorial disease, in which genetic effects are influenced by environmental and lifestyle factors and may not be generalized across populations [1,51,57]. In addition, key confounding factors, including diet, fluoride exposure, socioeconomic status, oral hygiene, oral microbiome composition, saliva, and access to dental care, were not consistently controlled for across the included studies. This may have influenced the reported genetic associations and contributed to the variability observed among studies. Overall, the current evidence does not yet support the implementation of genetic screening for dental caries risk in clinical practice, particularly given the influence of environmental and behavioral confounding factors.

Grouping genes based on their biological function provides a useful framework for organizing the literature on dental caries. However, this method simplifies a complex biological system, as many genes are multifunctional and participate in multiple pathways. For example, the *VDR* gene is involved in both mineralization and immune regulation [12]. Similarly, *ALOX15* has been reported in the literature within both TDM and IMR categories [7,27]. This overlap highlights the limitations of strict classification and the need for an integrated interpretation of genetic findings. However, this study analyzed each gene group as a cluster to reduce multiplicity and minimize false-positive associations.

### Limitations and Strengths

The quality of the available evidence should also be considered. Most of the included reviews were rated as low or critically low quality according to AMSTAR-2, which limits confidence in the reported associations. Consequently, the findings should be interpreted with caution, especially when associations are based on a small number of studies or exhibit substantial heterogeneity. Additionally, overlap of primary studies across reviews may have led to artificially inflated precision within these systematic reviews, as they did not account for the hierarchical structure of the data [24]. However, efforts were made to avoid overlap following the Cochrane Handbook [21] and to account for this dependency by considering the hierarchical structure in the analysis to provide reliable estimates of the associations. Another limitation of this umbrella review is the limited and inconsistent reporting of ethnicity across the included studies. This restricted our ability to conduct meaningful subgroup analyses by population group, despite evidence that genetic associations may vary across different ethnic backgrounds [58]. Some gene groups were evaluated based on a limited number of primary studies with small sample sizes. Therefore, future larger studies may provide more precise estimates and could potentially strengthen, weaken, or even change the direction of the observed associations. Although sensitivity analysis is recommended, it was not possible in this umbrella review because AMSTAR-2 assesses the quality of systematic reviews, whereas sensitivity analysis should be based on the quality of the primary studies, which was not assessed in this analysis.

Although certain variants showed an association with dental caries, further research is needed to move toward integrated approaches. Dental caries likely results from complex interactions between multiple genes and environmental and behavioral factors. Future studies should include larger and adequately powered cohorts to better detect modest genetic effects, along with more consistent and comparable measures of caries, such as the standardized use of DMFT/dmft and DMFS/dmfs indices (Decayed, Missing, and Filled Teeth and Surfaces indices for permanent and primary dentitions, respectively). Rather than focusing on single variants, future research should consider the combined influence of multiple genes and their interactions with the oral microbiome and environmental exposures. Such approaches may provide a more comprehensive understanding of dental caries risk, improve prevention strategies and enable personalized management. The nominally significant SNP associations identified in the present review should be regarded as preliminary, hypothesis-generating results. These findings require validation in larger, more diverse populations using standardized caries assessment methods and appropriate control of environmental confounding factors.

## 5. Conclusions

The evidence connecting genetic variants to dental caries susceptibility remains limited, inconsistent, and largely based on low-quality evidence. Although some associations with specific variants have been reported, most findings are not statistically significant after multilevel meta-analysis and should be interpreted with caution. Current evidence does not support the use of genetic markers for risk assessment or individualized prevention. Future studies should validate these findings in larger, well-characterized, diverse populations using standardized caries definitions and integrative gene–environment approaches while controlling for environmental and behavioral confounding factors.

## Figures and Tables

**Table 1:** Characteristics of included studies.

Author, Year	Design of Primary Studies	Age (Range)	Sample Size Range; (Total)	Independent Variables	Outcome Measures	Conclusion
Shojaei et al., 2026 [45]	CC	2–61 yrs	30–1354; (n = 11,789)	GPMs in *LTF*, *ENAM*, *AMELX*	DMFT/dmft and ICDAS	*LTF*, *ENAM*, and *AMELX* polymorphisms showed no consistent associations with dental caries susceptibility.
Zhou et al., 2025 [46]	GWAS	2–93 yrs	210–53,325	Genetic variants (SNPs)	DMFS/T, DFS/T, dfs/t, dmfs/t, dmftw (including WSL), DFSS, binary dental caries outcome, ICDAS, dfsPF, dfsSM, PF D1MFS, SM D1MFS	GWAS findings on dental caries were inconsistent due to study heterogeneity; only *NEDD9* (rs7738851) and *NAMPT* (rs190395159) were explored for association with dental caries in large studies.
Chisini et al., 2025 [41]	CC and CS	3–60 yrs	60–731; (n = 4944 in SR and 4425 in MA)	SNPs in *VDR*	DMFT, dmft, and ICDAS	No individual *VDR* SNP showed a significant association with dental caries.
Sharifi et al., 2021 [31]	CC	20 mo–60 yrs	81–355; (n = 1498)	GPMs in *CA6*, *AMBN*, *TUFT1*	DMFT/dmft	*CA6*, *AMBN*, and *TUFT1* polymorphisms showed no significant association with dental caries risk.
Nireeksha et al., 2021 [37]	CO	NR	4–400; (n = 994)	Salivary levels of LL-37 and GPMs in *VDR*	Dental caries	GPMs in *VDR* (Bsml and FokI) were associated with higher caries risk, while higher salivary LL-37 levels were associated with lower risk.
Molaei et al., 2022 [34]	CC and CS	3–65 yrs	81–893; (n = 4750)	GPMs in *MMP9*, *MMP13*, *MMP20*	DMFT/dmft	*MMP13* polymorphism was associated with reduced caries risk, while *MMP9* and *MMP20* showed no association.
Najafi-Ghobadi et al., 2023 [36]	CC	NR	81–782; (n = 2343)	GPMs in *MMP13*	Dental caries	No significant association was found between *MMP13* (rs2252070) polymorphism and caries susceptibility.
Motahari et al., 2024 [35]	CC and CS	5–45 yrs	182–1055; (n = 2978)	GPMs in *TAS1R2*	DMFT/dmft	*TAS1R2* (rs9701796) polymorphism increased caries risk. No association was found for (rs35874116).
Piekoszewska-Ziętek et al., 2017 [7]	NR	1–84 yrs	30–12,803; (n = 28,890)	SNPs in TDM (*BMP7*, *AMELX*, *ENAM*, *KLK4*, *TUFT1*, *ALOX15*, *KRT75*, *MMP20*, *AMBN*, *MMP13*, *DSPP*, *ESRRB*); SCF (*CA6*, *MUC7*, *AQP5*); IMR (*DEFB1*, *LTF*, *MBL2*, *MASP2*); and TPS (*TAS2R38*, *TAS1R2*, *TAS1R3*, *GLUT2*, *GNAT3*)	DMFT/DMFS and dmft/dmfs	*AMELX*, *AQP5*, and *ESRRB* were consistently linked to dental caries, indicating an important role in caries risk.
Cavallari et al., 2019 [20]	CC, CS and COH	2–51 yrs	50–1819; (n = 16,975)	*ACE I/D*, *ALOX15*, *AMBN*, *AMELX*, *APAL*, *AQP5*, *BMP2*, *CA6*, *CDX2*, *DB*, *DEFB1*, *DLX3*, *DSPP*, *ENAM*, *FOKL*, *FCN2*, *GLUT2*, *HLA-DR4*, *HLA-DQ2*, *HLA-DQ3*, *HLA-DQ4*, *HLA-DQ5*, *HLA-DQ6*, *KLK4*, *LTF*, *MASP2*, *MBL*, *MBL2*, *MG1*, *MG2*, *MMP2*, *MMP3*, *MMP9*, *MMP13*, *MMP20*, *MUC5B*, *OS*, *MUC7*, *PA*, *PB*, *PIF*, *PMF*, *PR*, *PRP1*, *SPP1*, *TAS1R2*, *TAS1R3*, *TAS2R38*, *TIMP1*, *TIMP2*, *TUFT1*, *TFIP11*, *VDR-TAQI*, *VDR-FOK*	DMFT/dmft	A total of 27 genes (*PRP1*, *PR*, *PA*, *MG1*, *MG2*, *AMELX*, *ENAM*, *TUFT1*, *KLK4*, *HLA-DR4*, *TAS1R3*, *TAS2R38*, *MBL2*, *MMP20*, *MMP2*, *MMP9*, *MMP13*, *GLUT2*, *TAS1R2*, *CA-VI*, *DEFB1*, *ALOX15*, *VDR-TAQI*, *MMP3*, *CA6*, *MUC5B*, *VDR-FOK*) were associated with either increased risk or protection against dental caries.
Hatipoglu et al., 2019 [13]	COH and CC	6–60 yrs	178–355; (n = 932)	SNPs in *CA6*	DMFT/dmft	No significant association was found between *CA6* polymorphisms and dental caries.
Sharifi et al., 2020 [30]	CC	1.7–60 yrs	71–1062; (n = 5432)	GPMs in *LTF*, *ENAM*, and *AMELX*	DMFT/dmft	Only *ENAM* (rs3796704) was associated with increased caries risk, particularly in Caucasians.
Li et al., 2020 [42]	CC	3–13 yrs	50–1005; (n = 3389)	Genetic variants in the *LTF*	DMFT and radiolucency	*LTF* variants (rs1126478) in moderate/severe cases and (rs1126477) were significantly associated with dental caries.
Hatipoğlu et al. 2020 [28]	CC	2–84 yrs	69–296; (n = 1419)	GPMs in *DEFB1*	DMFT/dmft	*DEFB1* (rs11362) polymorphism was significantly associated with caries in permanent dentition.
Ślebioda et al., 2021 [33]	CC	NR	69–4224; (n = 7200)	GPMs in *DEFB1*	Dental caries	Only rs1047031 polymorphism showed a significant association with caries susceptibility.
Li et al., 2021 [10]	CC	20 mo–60 yrs	71–1005; (n = 6589)	Genetic variants in TDM (*AMELX*, *ENAM*, *MMP2*, *MMP20*, *MMP13*, *AMBN*, *TUFT1*, *TFIP11*, *KLK4*, *MMP9*, *MMP3*, *MMP8*)	DMFT/S, dmft/s, dffs, and ICDAS	*AMELX*, *MMP20*, *MMP2* and *MMP13* variants were significantly associated with increased caries risk.
Sadeghi et al., 2021 [38]	CC	<18 yrs	120–549; (n = 2823)	GPMs in *VDR* (ApaI, Fokl, TaqI, BsmI and BglI)	Clinical examination of dental caries	No association with dental caries was found for most SNPs, except FokI (rs10735810).
Lei et al., 2021 [29]	CC	3–67 yrs	120–549; (n = 2470)	TaqI polymorphism in *VDR*	Dental caries	The C allele and CC genotype of the TaqI polymorphism were associated with increased caries risk, mainly in the permanent dentition in Asian.
Alkuhl et al., 2022 [40]	CS	Children 2–16 yrs Mean maternal age 18–45 yrs	38–600; (n = 3400)	Genetic taste sensitivity detected by (PROP)	DMFT/S, dmft/s and WSLs	Non-tasters of PROP showed significantly higher caries experience than medium and super tasters.
Hemati et al., 2023 [43]	CC	1–18 yrs	81–404; (n = 1850)	GPMs in *DEFB1* and *MBL2*	DMFT/dmft	*DEFB1* (rs11362) T allele was associated with increased caries risk in primary dentition.
Sharma et al., 2023 [11]	CC, COH and CS	20 mo–6 yrs	53–1005; (n = 4990)	SNPs in TDM (*AMELX*, *ENAM*, *TUFT1*, *MMP20*, *AMBN*, *KLK4*, *MMP9*, *MMP13*, *MMP2*, *MMP3*, *MMP10*, *MMP14*, *MMP16*, *TIMP1*, *TIMP2*, *TFIP11*)	ECC measured by dmft, deft and WSL	*AMBN*, *MMP9*, *MMP13*, *MMP20*, and *KLK4* polymorphisms were significantly associated with ECC.
Li et al., 2023 [44]	CC, COH and CS	20 mo–34 yrs	95–567; (n = 1311)	GPMs in *KLK4*	DMFT/dmft	*KLK4* (rs2235091) polymorphism was associated with caries susceptibility in primary, but not permanent dentition.
Aruna et al., 2023 [27]	CC, COH and CS	Children ≤6 yrs	37–1005; (n = 5399)	SNPs and genetic variants of IMR (*TRAV4*, *TNF-α*, *ALOX15*, *MBL2*, *DEFB1*, *LTF*, *LPO*, *HLA*, *MASP2*)	ECC primarily measured by the dmft	GPMs in *TNF-α*, *ALOX15*, *TRAV4*, and *HLA-DRB1* were associated with ECC susceptibility; *ALOX15* (rs7217186, TT) increased risk, while *LTF* (rs4547741, CT) was protective.
Qin et al., 2024 [47]	CC and CS	3–15 yrs	150–549; (n = 3517)	GPMs in *VDR* (ApaI, BsmI, TaqI, Fokl, TaqI/BglI, Cdx-2)	DMFT/dmft and ICDAS	FokI (rs10735810) and TaqI (rs731236) were associated with caries risk; TaqI was a risk factor for permanent dentition in Asian.
Lips et al., 2017 [32]	CT, CC, COH and CS	2–84 yrs	30–920	Salivary protein polymorphisms (*DEFB1*, *LTF*, *CA6*, *MUC7*, *PRH1* and *LYZL2*)	DMFT/dmft and DMFS	*DEFB1*, *LTF*, *CA6*, *MUC7*, *LYZL2*, and *PRH1* polymorphisms were associated with dental caries risk.
Chisini et al., 2020a [48]	CC, COH and CS	1–84 yrs	69–1005; (n = 6947)	SNPs in IMR (*MBL2*, *LFT*, *MASP2*, *DEFB1*, *FCN2*, *and MUC5B*)	DMFT/S, dmft/s, and ICDAS	*MBL2* and *MUC5B* were significantly associated with increased caries risk, while DEFB1 showed variable associations depending on the SNP.
Chisini et al., 2020b [49]	CC, COH and CS	1–72 yrs	96–3600; (n = 13,824)	SNPs in TDM (*AMBN*, *AMELX*, *BMP2*, *BMP4*, *BMP7*, *DLX3*, *ENAM*, *KLK4*, *MMP13*, *MMP2*, *MMP20*, *MMP3*, *MMP9*, *TFIP1*, *TIMP1*, *TIMP2*, *TUFT1*, *TFIP11*)	DMFT/S, dmft/s, WSLs and ICDAS	*TFIP11* and *AMELX* were linked to increased caries risk, whereas AMBN showed a protective effect.
Chisini et al., 2021 [39]	CC and COH	3–65 yrs	80–2249; (n = 4032)	SNPs in TPS (*TAS1R2*, *TAS2R38*, *TAS1R3*, *GLUT2*)	DMFT/S, dmft/s, and ICDAS	SNPs in TPS were associated with caries; *TAS2R38* (rs713598, CG) showed a protective effect.
Chisini et al., 2023 [50]	CC, COH and CS	3–72 yrs	43–1383; (n = 6207)	SNPs in SCF (*CA6*, *AQP2*, *AQP5*, *MUC5B*)	DMFT/S, dmft/s, WSLs, and ICDAS	*CA6*, *AQP2* and *AQP5* were associated with dental caries experience.

**Abbreviations:** CC: Case–Control Studies; GWAS: Genome-Wide Association Studies; CS: Cross-Sectional Studies; CO: Controlled Observational Studies; COH: Cohort Studies; CT: Clinical Trials; mo: months; yrs: years; Country abbreviations are based on ISO 3166-1 alpha-3 codes; SNPs: Single-nucleotide polymorphisms; GPMs: Genetic Polymorphisms; TDM: Tooth Development and Mineralization Genes; SCF: Salivary Composition and Function Genes; IMR: Immune and Inflammatory Responses Genes; TPS: Taste Perception and Signaling Genes; VDR: Vitamin D Receptor Genes; ECC: Early Childhood Caries.

**Table 2:** Multilevel meta-analysis of genetic polymorphisms associated with dental caries.

Gene Category	Gene	SNP	Adjusted OR (95% CI)	*p*-Value
Tooth Development and Mineralization	Pooled effect		1.01 (0.86–1.17)	0.93
	*ENAM*	rs12640848	1.06 (0.55–2.05)	0.85
		rs1264848	0.94 (0.48–1.84)	0.85
		rs3796703	1.09 (0.54–2.19)	0.81
		rs3796704	1.22 (0.61–2.45)	0.58
	*MMP9*	rs17576	0.93 (0.47–1.85)	0.83
	*MMP20*	rs1784418	0.91 (0.48–1.71)	0.76
	*AMELX*	rs17878486	1.55 (0.79–3.03)	0.20
		rs2106416	0.92 (0.33–2.6)	0.88
	*KLK4*	rs2235091	1.22 (0.63–2.39)	0.56
	*MMP13*	rs2252070	0.81 (0.42–1.54)	0.52
	*TFIP11*	rs5997096	0.74 (0.31–1.77)	0.49
	Genetic models and genotypes	Allelic	Ref	
		Dominant	1.01 (0.88–1.15)	0.92
		Heterozygote	0.99 (0.86–1.13)	0.83
		Homozygous	0.99 (0.87–1.14)	0.93
		Recessive	0.97 (0.84–1.12)	0.68
		Wild	0.82 (0.63–1.07)	0.14
	*VDR*	Pooled effect	0.96(0.84–1.11)	0.62
		rs10735810 (FokI)	0.92 (0.66–1.29)	0.62
		rs11568820 (Cdx2)	0.66 (0.46–0.95)	0.03
		rs1544410 (BsmI)	0.92 (0.63–1.36)	0.68
		rs2228570 (FokI)	1.18 (0.8–1.75)	0.40
		rs731236 (TaqI)	1.00 (0.73–1.37)	0.98
		rs739837 (3′ UTR var)	1.00 (0.71–1.4)	0.99
		rs7975232 (ApaI)	0.96 (0.69–1.32)	0.78
	Genetic models and genotypes (VDR)	Allelic	Ref	
		Dominant	0.92 (0.73–1.16)	0.49
		Heterozygous	1.25 (1–1.57)	0.05
		Homozygous	1.20 (0.88–1.63)	0.24
		Over-dominant	1.17 (1–1.37)	0.04
		Recessive	1.05 (0.91–1.2)	0.53
Salivary Composition and Function	Pooled effect		1.07(0.92–1.25)	0.38
	*AQP2*	rs10864376	0.8 (0.46–1.41)	0.42
		rs12138897	1.8 (0.88–3.66)	0.10
		rs12021597	1.9 (0.88–4.11)	0.10
	*MUC5B*	rs2735733	0.94 (0.57–1.57)	0.81
		rs2857476	1.12 (0.66–1.89)	0.66
		rs2249073	1.11 (0.66–1.89)	0.67
	*CA6*	rs2274327	0.95 (0.56–1.61)	0.84
		rs2274328	0.94 (0.52–1.68)	0.82
		rs2274333	0.93 (0.57–1.51)	0.75
	*AQP5*	rs10875989	1.39 (0.84–2.28)	0.19
		rs17032907	1.48 (0.77–2.86)	0.23
		rs923911	1.02 (0.57–1.83)	0.96
		rs3759129	0.70 (0.41–1.19)	0.17
		rs1996315	0.92 (0.52–1.61)	0.75
	Genetic models and genotypes	Allelic	Ref	
		Heterozygous	0.99 (0.77–1.28)	0.95
		Homozygous	1.26 (0.92–1.72)	0.14
Immune and Inflammatory Responses	Pooled effect		1.06(0.96–1.17)	0.24
	*LTF*	rs1126478	1.04 (0.71–1.52)	0.83
	*DEFB1*	rs11362	1.2 (0.89–1.62)	0.22
		rs1799946	0.9 (0.66–1.23)	0.51
		rs1800972	1.26 (0.65–2.46)	0.49
	*MBL2*	rs1800450	1.48 (1.03–2.14)	0.04
		rs7096206	0.95 (0.66–1.37)	0.78
	Genetic models and genotypes	Allelic	Ref	
		Dominant	1.01 (0.82–1.25)	0.92
		Heterozygous	0.99 (0.81–1.22)	0.96
		Homozygous	0.94 (0.72–1.21)	0.62
		Recessive	0.95 (0.76–1.21)	0.67
		Wild	1.01 (0.72–1.43)	0.95
Taste Perception and Signaling	Pooled effect		0.57 (0.15–2.16)	0.40
	*TAS1R2*	rs35874116	1.04 (0.5–2.16)	0.91
	*TAS2R38*	rs713598	0.26 (0.09–0.73)	0.01
	*TAS1R2*	rs9701796	1.13 (0.54–2.37)	0.74
	Genetic models and genotypes	Allelic	Ref	
		Dominant	0.95 (0.79–1.15)	0.61
		Heterozygous	0.92 (0.76–1.12)	0.41
		Homozygous	1.34 (0.94–1.9)	0.10
		Recessive	1.39 (1–1.95)	0.05

## Data Availability

This umbrella review utilized data extracted from previously published systematic reviews and meta-analyses. All data analyzed during this study are included in this article and its Supplementary Information Files.

## Supplementary Materials

The following supporting information can be downloaded at: https://www.mdpi.com/article/10.3390/genes17060724/s1, Supplementary File S1: PRISMA 2020 checklist; Supplementary File S2: Documentation of search strategies; Supplementary File S3: AMSTAR 2 checklist; Supplementary Table S1: Primary study overlap across reviews; Supplementary Table S2: Jadad-based review selection; Supplementary Table S3: Excluded studies and reasons for exclusion; Supplementary Table S4: Summary of genes, single-nucleotide polymorphisms (SNPs), chromosomal locations, and functional classification; Supplementary Table S5: Detailed characteristics of included studies. References [7,10,11,13,20,27,28,29,30,31,32,33,34,35,36,37,38,39,40,41,42,43,44,45,46,47,48,49,50,52,54,59,60,61,62,63,64,65,66,67,68,69,70,71,72,73,74,75,76,77,78,79,80,81,82,83,84,85,86,87,88,89,90,91,92,93,94,95,96,97,98,99,100,101,102,103,104,105,106,107,108,109,110,111,112,113,114,115,116,117,118,119,120,121,122,123,124,125,126,127,128] are cited in the Supplementary Materials.

## Abbreviations

The following abbreviations are used in this manuscript:

SNPs	Single-nucleotide polymorphisms
TDM	Tooth development and mineralization
SCF	Salivary composition and function
IMR	Immune and inflammatory responses
TPS	Taste perception and signaling
AMELX/AMELY	Amelogenin
ENAM	Enamelin
AMBN	Ameloblastin
TUFT1	Tuftelin 1
KLK4	Kallikrein-related peptidase 4
MMP2	Matrix metallopeptidase 2
MMP9	Matrix metallopeptidase 9
MMP13	Matrix metallopeptidase 13
MMP20	Matrix metallopeptidase 20
DSPP	Dentin sialophosphoprotein
VDR	Vitamin D receptor
CA6	Carbonic anhydrase VI
AQP5	Aquaporin 5
MUC7	Mucin 7
MUC5B	Mucin 5B
DEFB1	Beta-defensin 1
LTF	Lactoferrin
MBL2	Mannose-binding lectin 2
MASP2	MBL-associated serine protease 2
TAS1R2	Taste receptor type 1 member 2
TAS1R3	Taste receptor type 1 member 3
TAS2R38	Taste receptor type 2 member 38
PRISMA	Preferred Reporting Items for Systematic Reviews and Meta-Analyses
MeSH	Medical Subject Headings terms
AMSTAR-2	A Measurement Tool to Assess Systematic Reviews, version 2
Q-Genie	Quality of Genetic Association Studies tool
JBI	Joanna Briggs Institute Critical Appraisal Checklist
GRADE	Grading of Recommendations Assessment, Development and Evaluation
NOS	Newcastle–Ottawa Scale
AHRQ	Agency for Healthcare Research and Quality
CC	Case–Control Studies
GWAS	Genome-Wide Association Studies
CS	Cross-Sectional Studies
CO	Controlled Observational Studies
COH	Cohort Studies
CT	Clinical Trials
GPMs	Genetic Polymorphisms
ECC	Early Childhood Caries
NR	Not Reported
NA	Not Assessed
NM	No Meta-analysis
