# Supplementary material for: Genetic Variants and Dental Caries Susceptibility: An Umbrella Review and Multilevel Meta-Analysis
Source: Genes (Basel). 2026 Jun 22;17(6):724. doi: 10.3390/genes17060724 (PMC13299435; doi:10.3390/genes17060724)
Supplement: Supplementary file 1 [file genes-17-00724-s001.zip › Supplementary File S2.pdf]

Biomedical Library, Gothenburg University Library

Documentation of search strategies

Date: 2024-12-17

Topic/research question:
Genes associated with dental caries: an umbrella review and meta-analysis

Name of researchers: Halah Khalifa

Librarians: Eva Hessman & Helen Sjöblom

Databases: Pubmed, Cochrane Library, Embase, Web of Science, Scopus, Virtual Health Library

---

Total number of hits:

Before deduplication: n = 505
After deduplication: n = 263

Updated search:

Date: 2026-02-25
Additional number of hits: n = 44 after deduplication (total n = 502)

Comments:
LILACS not available for search on the day of update searching
"Searches on the VHL may experience temporary instability due to adjustments in security rules. If you need assistance, please contact us."
Provider contacted by e-mail 25 feb 2026

PubMed

Interface: pubmed.gov<br><br>Date of Search: 2024-12-17<br><br>Number of hits: 78<br><br>Comment: ~2 proximity operator, within 2 words, regardless of order	Field labels<br><br>• [mh] or [mesh] = exploded MeSH term<br>• [tiab] = title, abstract<br>• [tw] = textwords: all words and numbers in the title, abstract, other abstract, MeSH terms, MeSH Subheadings, Publication Types, Substance Names<br>* = truncation of word for alternate endings<br>NB If truncating a phrase – the truncated term must be the last word in the phrase.

#	Searches	Results
4	#1 AND #2 AND #3	78
3	(Systematic Review[Publication Type] OR Systematic Reviews as Topic[mh] OR meta-analysis[pt] OR meta-analysis as topic[mh] OR meta-analysis[mh] OR "systematic review"[tiab:~2] OR "systematic reviews"[tiab:~2] OR "systematic overview"[tiab:~2] OR "systematic overviews"[tiab:~2] OR "meta analysis"[tiab:~2] OR "meta analyses"[tiab:~2] OR metanaly*[tw] OR metaanaly*[tw] OR met analy*[tw] OR meta-synthes* OR metasynthes*)	560865
2	(gene[tiab] OR genes[tiab] OR genetic[tiab] OR genetics[tiab] OR Genome wide association study[mh] OR GWAS[tiab] OR Polymorphism, single nucleotide[mh] OR polymorphism, genetic[mh] OR polymorphism*[tiab] OR heredit*[tiab] OR genetics[Subheading])	5470797
1	(caries[tiab] OR carious[tiab] OR dental caries[mh] OR dental decay[tiab] OR dental cavit*[tiab] OR dental caries susceptibility[mh])	73762

Scopus

Interface: Elsevier<br><br>Date of Search: 2024-12-17<br><br>Number of hits: 147<br><br>Comment:	Field labels<br><br>• TITLE = title<br>• ABS = abstract<br>• KEY = keywords<br>• W/x = within x words, regardless of order<br>• * = truncation of word for alternate endings

#	Searches	Results
4	1 AND 2 AND 3	147
3	TITLE-ABS-KEY ((systematic W/2 review) OR (systematic W/2 reviews) OR (systematic W/2 overview) OR (systematic W/2 overviews) OR (meta W/2 analysis) OR (meta W/2 analyses) OR metanaly* OR metaanaly OR "met analy*" OR "meta-synthes*" OR metasynthes)	833698
2	TITLE-ABS-KEY(gene OR genes OR genetic OR genetics OR GWAS OR polymorphism* OR heredit*)	7117663
1	TITLE-ABS-KEY(caries OR carious OR "dental decay" OR "dental cavit*")	99270

Cochrane Library

Interface: Cochrane Library<br><br>Date of Search: 2024-12-16<br><br>Number of hits: 6<br><br>Comment: If using wildcards, use NEXT operator, phrase search does not support the use of wildcards.	Field labels<br><br>• ti,ab,kw = title, abstract and author keywords<br>• NEAR/x = within x words, regardless of order<br>• * = truncation of word for alternate endings

ID	Search	Hits
#1	MeSH descriptor: [Systematic Reviews as Topic] explode all trees	134
#2	MeSH descriptor: [Systematic Review] explode all trees	1
#3	("systematic review"):pt	0
#4	MeSH descriptor: [Meta-Analysis as Topic] explode all trees	1633
#5	("meta analysis"):pt	0
#6	(systematic near/2 review):ti,ab,kw	9756
#7	(systematic near/2 overview):ti,ab,kw	114
#8	(meta near/2 analysis):ti,ab,kw	22916
#9	(metanaly*):ti,ab,kw	126
#10	(metaanaly*):ti,ab,kw	17414
#11	(met next analy*):ti,ab,kw	48
#12	("metasynthesis"):ti,ab,kw	8
#13	("meta-synthesis"):ti,ab,kw	5
**#14**	#1 OR #2 OR #3 OR #4 OR #5 OR #6 OR #7 OR #8 OR #9 OR #10 OR #11 OR #12 OR #13	28767
#15	MeSH descriptor: [Dental Caries] explode all trees	3853
#16	MeSH descriptor: [Dental Caries Susceptibility] explode all trees	279
#17	(caries OR carious OR "dental decay"):ti,ab,kw	10601
#18	(dental next cavit*):ti,ab,kw	866
**#19**	#15 OR #16 OR #17 OR #18	11058
#20	MeSH descriptor: [Genome-Wide Association Study] explode all trees	856
#21	MeSH descriptor: [Polymorphism, Single Nucleotide] explode all trees	2561
#22	MeSH descriptor: [Polymorphism, Genetic] explode all trees	4852
#23	MeSH descriptor: [] explode all trees and with qualifier(s): [genetics - GE]	26255
#24	(gene OR genetic OR genetics OR GWAS OR polymorphism OR heredit*):ti,ab,kw	82793
**#25**	#20 OR #21 OR #22 OR #23 OR #24	82804
**#26**	#14 AND #19 AND #25	6
**#27**	Filter: in reviews	0

Web of Science Core Collection

Interface: Clarivate Analytics<br><br>Date of Search: 2024-12-17<br><br>Number of hits:80	Field labels<br><br>• TS/Topic = title, abstract, author keywords and Keywords Plus<br>• NEAR/x = within x words, regardless of order<br>• * = truncation of word for alternate endings

#	Searches	Results
4	1 AND 2 AND 3	80
3	TS=((systematic NEAR/2 review) OR (systematic NEAR/2 reviews) OR (systematic NEAR/2 overview) OR (systematic NEAR/2 overviews) OR (meta NEAR/2 analysis) OR (meta NEAR/2 analyses) OR metanaly* OR metaanaly OR “met analy*” OR “meta-synthes*” OR metasynthes*)	627676
2	TS=(gene OR genes OR genetic OR genetics OR GWAS OR polymorphism* OR heredit*)	4909817
1	TS=(caries OR carious OR “dental decay” OR “dental cavit*”)	54331

Embase

Interface: Elsevier<br><br>Date of Search: 2024-12-17<br><br>Number of hits: 108<br><br>Comment: use ' for phrase searching	Field labels<br><br>• /exp = exploded Emtree term<br>• /de = non exploded Emtree term<br>• :ti,ab,kw. = title, abstract and author keywords<br>• NEAR/n = within x words, regardless of order<br>• * = truncation of word for alternate endings<br>• * = variable wildcard, i.e one or more letters<br>• ' = phrase

#	Searches	Results
10	3 AND 6 AND 9	108
9	7 OR 8	792685
8	(systematic NEAR/2 review OR systematic NEAR/2 reviews OR systematic NEAR/2 overview OR systematic NEAR/2 overviews OR meta NEAR/2 analysis OR meta NEAR/2 analyses OR metanaly* OR metaanaly OR 'met analy*' OR 'meta-synthes*' OR metasynthes*):ti,ab,kw	635753
7	'systematic review'/exp OR 'meta analysis'/exp OR 'meta synthesis'/exp	636875
6	4 OR 5	4513379
5	(gene OR genes OR genetic OR genetics OR GWAS OR polymorphism* OR heredit*):ti,ab,kw	4409363
4	'genome-wide association study'/exp OR 'single nucleotide polymorphism'/exp OR 'genetic polymorphism'/exp	551394
3	1 OR 2	81856
2	(caries OR carious OR 'dental decay' OR 'dental cavit*'):ti,ab,kw	60886
1	'dental caries'/exp	67193

LILACS – Virtual Health Library

<table>
<tr><td>Interface: https://bvsalud.org/en/

Date of Search: 2024-12-18

Number of hits: 86

Comment: inte samma sökmöjligheter som i övriga databaserna, så söksträngen är förenklad</td><td></td></tr>
<tr><td colspan="2">(caries OR carious OR "dental decay" OR "dental cavit*") AND (gene* OR genome* OR gwas OR polymorphism OR heredit*)
→ 2,777

Filter: Type of study: Systematic review, Systematic review of observational studies, Structured summary of systematic review
→ 86</td></tr>
</table>
