# Supplementary material for: Genetic Variants and Dental Caries Susceptibility: An Umbrella Review and Multilevel Meta-Analysis
Source: Genes (Basel). 2026 Jun 22;17(6):724. doi: 10.3390/genes17060724 (PMC13299435; doi:10.3390/genes17060724)
Supplement: Supplementary file 1 [file genes-17-00724-s001.zip › Supplementary Table S1.pdf]

Supplementary Table S1. Primary Study Overlap Across Systematic Reviews and Selection of Data Sources for Each SNP Included in the Meta-Analysis.

Gene	SNP	No. of Studies	Studies	Outcome measuremnet
AMELX	rs17878486	7 Priamry Studies	Ergoz et al., 2014; Gachova et al., 2023; Gerreth et al., 2017; Gerreth et al., 2017; Jeremias et al., 2013; Kang et al., 2011; Khami et al., 2022 [14, 16, 18, 24, 25, 35]	Ergoz et al., 2014: DMFT/dmft and DMFS/dmfs<br>Gachova et al., 2023: DMFT<br>Gerreth et al., 2017: Cavitated lesions (dt) and incipient caries (di)<br>Jeremias et al., 2013: DMFT/dmft<br>Kang et al., 2011: DMFT and DMFS were evaluated<br>Khami et al., 2022: DMFT index
AMELX	rs2106416	SR	Sharifi et al., 2020 [48]	DMFT/dmft
AQP2	rs10875989	SR	Chisini et al., 2023 [49]	DMFT/S, dmft/s, WSLs, and ICDAS
AQP5	rs1996315	SR	Chisini et al., 2023 [49]	DMFT/S, dmft/s, WSLs, and ICDAS
AQP5	rs3759129	SR	Chisini et al., 2023 [49]	DMFT/S, dmft/s, WSLs, and ICDAS
AQP5	rs923911	SR	Chisini et al., 2023 [49]	DMFT/S, dmft/s, WSLs, and ICDAS
ApaI	rs7975232	SR	Chisini et al., 2025 [50]	DMFT, dmft, and ICDAS
BsmI	rs1544410	SR	Chisini et al., 2025 [50]	DMFT, dmft, and ICDAS
CA6	rs10864376	SR	Chisini et al., 2023 [49]	DMFT/S, dmft/s, WSLs, and ICDAS
CA6	rs12021597	SR	Chisini et al., 2023 [49]	DMFT/S, dmft/s, WSLs, and ICDAS

CA6	rs12138897	SR	Chisini et al., 2023 [49]	DMFT/S, dmft/s, WSLs, and ICDAS
CA6	rs17032907	SR	Chisini et al., 2023 [49]	DMFT/S, dmft/s, WSLs, and ICDAS
CA6	rs2274327	SR	Chisini et al., 2023 [49]	DMFT/S, dmft/s, WSLs, and ICDAS
CA6	rs2274328	SR	Chisini et al., 2023 [49]	DMFT/S, dmft/s, WSLs, and ICDAS
CA6	rs2274333	SR	Chisini et al., 2023 [49]	DMFT/S, dmft/s, WSLs, and ICDAS
Cdx2	rs11568820	SR	Chisini et al., 2025 [50]	DMFT, dmft, and ICDAS
DEFB1	rs11362	4 Priamry Studies	Abbasoglu et al., 2015; de Oliveira et al., 2018; Lips et al., 2017; Mubayrik et al., 2014 [1, 6, 51, 52]	Abbasoglu et al., 2015: dmft<br>de Oliveira et al., 2018: dmft and DMFT index;<br>Lips et al., 2017: DMFT/dmft and DMFS<br>Mubayrik et al., 2014: DMFT or DMFS
DEFB1	rs1799946	2 Priamry Studies	de Oliveira et al., 2018; Lips et al., 2017 [51, 52]	de Oliveira et al., 2018: dmft and DMFT index;<br>Lips et al., 2017: DMFT/dmft and DMFS
DEFB1	rs1800972	2 Priamry Studies	Abbasoglu et al., 2015; Mubayrik et al., 2014 [1, 6]	Abbasoglu et al., 2015: dmft<br>Mubayrik et al., 2014: DMFT or DMFS
ENAM	rs12640848	6 Priamry Studies	Gerreth et al., 2016; Linhartova et al., 2016; Linhartova et al., 2016 Permanent; Linhartova et	Gerreth et al., 2016: Cavitated lesions

			al., 2016 Primary; Linhartova et al., 2018; Pang et al., 2021 [8, 9, 17, 32]	(dt) and incipient caries (di)<br><br>Linhartova et al., 2016: DMFT and dmft<br><br>Linhartova et al., 2018: DMFT and dmft<br><br>Pang et al., 2021: DMFT and ICDAS
ENAM	rs1264848	5 Priamry Studies	Divakar et al., 2019; Ergoz et al., 2014; Gerreth et al., 2016; Jeremias et al., 2013; Linhartova et al., 2018 [8, 13, 14, 17, 24]	Divakar et al., 2019: DMFT/DMFS and Dft/dfs<br><br>Ergoz et al., 2014: DMFT/dmft and DMFS/dmfs<br><br>Gerreth et al., 2016: Cavitated lesions (dt) and incipient caries (di)<br><br>Jeremias et al., 2013: DMFT/dmft<br><br>Linhartova et al., 2018: DMFT and dmft
ENAM	rs3796703	4 Priamry Studies	Negm 2023; Negm et al., 2024; Pang et al., 2021; Wang et al., 2017 [30, 31, 32, 42]	Negm 2023: DMFT<br><br>Negm et al., 2024: DMF-T and ICDAS-II<br><br>Pang et al., 2021: DMFT and ICDAS<br><br>Wang et al., 2017: dmft
ENAM	rs3796704	4 Priamry Studies	Divakar et al., 2019; Ergoz et al., 2014; Jeremias et al., 2013; Koohpeima et al., 2018 [13, 14, 24, 53]	Divakar et al., 2019: DMFT/DMFS and Dft/dfs<br><br>Ergoz et al., 2014: DMFT/dmft and DMFS/dmfs

				Jeremias et al., 2013: DMFT/dmft<br>Koohpeima et al., 2018: DMFT
FokI	rs10735810	SR	Chisini et al., 2025 [50]	DMFT, dmft, and ICDAS
FokI	rs2228570	SR	Qin et al., 2024 [54]	DMFT/dmft and ICDAS
KLK4	rs2235091	5 Priamry Studies	Abbasoğlu et al., 2015; Cavallari et al., 2017; Gachova et al., 2023; Gachova 2023; Zaorskav et al., 2021 [1, 11, 16, 47]	Abbasoğlu et al., 2015: dmft<br>Cavallari et al., 2017: ICDAS<br>Gachova 2023: DMFT/dmft<br>Zaorskav et al., 2021: DMFT
LTF	rs1126478	5 Priamry Studies	Azevedo et al., 2010; Pang et al., 2021; Volckova et al., 2014; Wang et al., 2017; Wang et al., 2018 [5, 32, 40, 41, 42]	Azevedo et al., 2010: DMFT<br>Pang et al., 2021: DMFT and ICDAS<br>Volckova et al., 2014: dmft<br>Wang et al., 2017: dmft<br>Wang et al., 2018: dmft
MBL2	rs1800450	3 Priamry Studies	Chisini 2020a; Pehlivan 2005; Yang 2013 [33, 43, 55]	Chisini et al., 2020a: DMFT/S, dmft/s, and ICDAS<br>Pehlivan et al., 2005: dmft<br>Yang et al., 2013: dmft
MBL2	rs7096206	3 Priamry Studies	Alyousef et al., 2017; Hu et al., 2020; Shimomura-Kuroki et al., 2018 [2, 22, 36]	Alyousef et al., 2017: DMFT<br>Hu et al., 2020: DMFT

				Shimomura-Kuroki et al., 2018: DMFT/dmft
MMP13	rs2252070	6 Priamry Studies	Dindaroglu 2021; Hu 2019; Linhartova 2020; Shimomura 2018; Tannure 2012; Vasconcelos 2019 [7, 10, 21, 36, 37, 38, 39]	Dindaroglu et al., 2021: DMFT and dmft<br><br>Hu et al. 2019: DMFT<br><br>Linhartova et al., 2020: DMFT and dmft<br><br>Shimomura et al., 2018: DMFT<br><br>Tannure et al., 2012: dmft/DMFT<br><br>Vasconcelos et al., 2019: dmft and DMFT index
MMP20	rs1784418	12 Priamry Studies	Antunes et al. 2016; Antunes et al., 2016; Dindaroglu et al., 2021; Filho et al., 2016; Gerreth et al., 2017; Gerreth et al., 2017; Kelly et al., 2020; Linhartova et al., 2020; Tannure et al., 2012a; Tannure et al., 2012b; Vasconcelos et al., 2019; Vasconcelos et al., 2019 [3, 7, 10, 15, 18, 26, 37, 38, 39]	Antunes et al., 2016: WSL and dmft<br><br>Dindaroglu et al., 2021: DMFT and dmft<br><br>Filho et al., 2016: dmft<br><br>Gerreth et al., 2017: Cavitated lesions (dt) and incipient caries (di)<br><br>Kelly et al., 2020: extreme caries phenotype<br><br>Linhartova et al., 2020: DMFT and dmft<br><br>Tannure et al., 2012a: dmft/DMFT<br><br>Tannure et al., 2012b: dmft/DMFT

				Vasconcelos et al., 2019: dmft and DMFT index
MMP9	rs17576	3 Priamry Studies	Antunes et al., 2016; Linhartova et al., 2020; Tannure et al., 2012 [3, 7, 37, 38]	Antunes et al., 2016: dmft and WSL<br>Linhartova et al., 2020: DMFT/dmft<br>Tannure 2012 et al.,: DMFT/dmft
MUC5B	rs2249073	SR	Chisini et al., 2023 [49]	DMFT/S, dmft/s, WSLs, and ICDAS
MUC5B	rs2735733	SR	Chisini et al., 2023 [49]	DMFT/S, dmft/s, WSLs, and ICDAS
MUC5B	rs2857476	SR	Chisini et al., 2023 [49]	DMFT/S, dmft/s, WSLs, and ICDAS
TAS1R2	rs35874116	6 Priamry Studies	Chisini et al. 2021; Haznedaroghlu et al. 2015; Izacovicova Holla et al. 2015; Liang et al. 2022; Wang et al. 2017; Yilmaz et al. 2021 [19, 20, 29, 42, 45, 56]	Chisini et al. 2021: DMFT/S, dmft/s, and ICDAS<br>Haznedaroghlu et al. 2015: dft/DMFT<br>Izacovicova Holla et al. 2015: DMFT<br>Liang et al. 2022: dmft<br>Wang et al., 2017: dmft<br>Yilmaz et al. 2021: DMFT
TAS1R2	rs9701796	3 Priamry Studies	Haznedaroghlu et al. 2015; Wang et al. 2017; Yilmaz et al. 2021 [19, 42, 45]	Haznedaroghlu et al. 2015: dft/DMFT<br>Wang et al., 2017: dmft<br>Yilmaz et al. 2021: DMFT

TAS2R38	rs713598	2 Priamry Studies	Shimomura et al., 2018; Yildiz et al., 2016 [36, 44]	Shimomura et al., 2018: DMFT<br>Yildiz et al., 2016: DMFT
TFIP11	rs5997096	SR	Chisini et al., 2020b [57]	DMFT/S, dmft/s, WSLs and ICDAS
TaqI	rs731236	7 Priamry Studies	Aribam et al., 2020; Chisini et al., 2025; Cogulu et al., 2016; Holla et al., 2017; Kong et al., 2017; Qin et al., 2019 ; Yu et al., 2017 [4, 12, 23, 27, 34, 46, 50]	Aribam et al., 2020: DMFT<br>Chisini et al., 2025: DMFT, dmft, and ICDAS<br>Cogulu et al., 2016: DMFT, dft<br>Holla et al., 2017: DMFT<br>Kong et al., 2017: dmft<br>Qin et al., 2019: dmft<br>Yu et al., 2017: DMFT
TaqI	rs739837	SR	Qin et al., 2024 [54]	DMFT/dmft and ICDAS

References:

1. Abbasoɫlu, Z., Tanboɫa, I., Küchler, E.C., Deeley, K., Weber, M., Kaspar, C., Korachi, M., Vieira, A.R.. Early childhood caries is associated with genetic variants in enamel formation and immune response genes. *Caries Res*, **2015**, *49*, 70–77. https://doi.org/10.1159/000362825
2. Alyousef, Y.M., Borgio, J.F., Abdulazeez, S., Al-Masoud, N., Al-Ali, A.A., Al-Shwaimi, E., Al-Ali, A.K.. Association of MBL2 Gene Polymorphism with Dental Caries in Saudi Children. *Caries Res*, **2017**, *51*, 12–16. https://doi.org/10.1159/000450963
3. Antunes, L.A., Antunes, L.S., Küchler, E.C., Lopes, L.B., Moura, A., Bigonha, R.S., Abreu, F.V., Granjeiro, J.M., de Amorim, L. M. da F., Paixão, I.C.N.P. Analysis of the association between polymorphisms in MMP2, MMP3, MMP9, MMP20, TIMP1, and TIMP2 genes with white spot lesions and early childhood caries. *Int. J. Paediatr. Dent.* **2016**, *26*, 310–319. https://doi.org/10.1111/IPD.12202
4. Aribam, V., Aswath, N., & Ramanathan, A. Single-nucleotide polymorphism in Vitamin D receptor gene and its association with dental caries in children. *J. Indian Soc. Pedod. Prev. Dent.* **2020**, *38*, 8–13. https://doi.org/10.4103/JISPPD.JISPPD_222_19
5. Azevedo, L.F., Pecharki, G.D., Brancher, J.A., Cordeiro Junior, C.A., Medeiros, K.G. dos S., Antunes, A.A., Arruda, E.S., Werneck, R.I., de Azevedo, L.R., Mazur, R.F., Moysés, S.J., Moysés, S.T., Faucz, F.R., & Trevilatto, P.C. Analysis of the association between lactotransferrin (LTF) gene polymorphism and dental caries. *J. Appl. Oral Sci.* **2010**, *18*, 166–170. https://doi.org/10.1590/S1678-77572010000200011
6. Bin Mubayrik, A., Deeley, K., Patir, A., Koruyucu, M., Seymen, F., Vieira, A.R. Polymorphisms In The Antimicrobial Peptide Defb1 Are Not Associated With Caries In Primary Dentition. *Journal of The Pakistan Dental Association.* **2014**, *23*. https://www.jpda.com.pk/public/polymorphisms-in-the-antimicrobial-peptide-defb1-are-not-associated-with-caries-in-primary-dentition
7. Borilova Linhartova, P., Deissova, T., Kukletova, M., Izakovicova Holla, L. Matrix metalloproteinases gene variants and dental caries in Czech children. *BMC Oral Health*. **2020**, *20*, 138. https://doi.org/10.1186/S12903-020-01130-6
8. Borilova Linhartova, P., Deissova, T., Musilova, K., Zackova, L., Kukletova, M., Kukla, L., Izakovicova Holla, L. Lack of association between ENAM gene polymorphism and dental caries in primary and permanent teeth in Czech children. *Clin. Oral Invest*. **2017**, *22*, 1873–1877. https://doi.org/10.1007/S00784-017-2280-2
9. Borilova Linhartova, P., Kastovsky, J., Bartosova, M., Musilova, K., Zackova, L., Kukletova, M., Kukla, L., Izakovicova Holla, L. ACE Insertion/Deletion Polymorphism Associated with Caries in Permanent but Not Primary Dentition in Czech Children. *Caries Res.* **2016**, *50*, 89–96. https://doi.org/10.1159/000443534
10. Çağırır Dindaroğlu, F., Eronat, N., Durmaz, A., Çoğulu, D., Durmaz, B., Çoğulu, Ö. The association between genetic polymorphisms in matrix metalloproteinases and caries experience. *Clin. Oral Invest.* **2021**, *25*, 5403–5410. https://doi.org/10.1007/S00784-021-03848-1
11. Cavallari, T., Tetu Moyses, S., Moyses, S.J., Iani Werneck, R. KLK4 Gene and Dental Decay: Replication in a South Brazilian Population. *Caries Res.* **2017**, *51*, 240–243. https://doi.org/10.1159/000464450
12. Cogulu, D., Onay, H., Ozdemir, Y., Aslan, G.I., Ozkinay, F., Eronat, C. The Role of Vitamin D Receptor Polymorphisms on Dental Caries. *The Journal of Clinical Pediatric Dentistry*. **2016**, *40*, 211–214. https://doi.org/10.17796/1053-4628-40.3.211
13. Devang Divakar, D., Alanazi, S.A.S., Assiri, M.Y.A., Mohammed Halawani, S., Zaid Alshehri, S., Ahmed Saeed Al-Amri, S., Mustafa, M. Association between ENAM polymorphisms and dental caries in children. *Saudi J. of Biol. Sci*. **2019**, *26*, 730–735. https://doi.org/10.1016/J.SJBS.2018.01.010
14. Ergöz, N., Seymen, F., Gencay, K., Tamay, Z., Deeley, K., Vinski, S., Vieira, A.R. Genetic Variation in Ameloblastin Is Associated with Caries in Asthmatic Children. *Eur. Arch. Paediatr. Dent*. **2013**, *15*, 211. https://doi.org/10.1007/S40368-013-0096-6
15. Filho, A.V.A., Calixto, M.S., Deeley, K., Santos, N., Rosenblatt, A., Vieira, A.R. MMP20 rs1784418 Protects Certain Populations against Caries. *Caries Res*. **2017**, *51*, 46–51. https://doi.org/10.1159/000452345
16. Gachova, D., Lipovy, B., Deissova, T., Izakovicova Holla, L., Danek, Z., Borilova Linhartova, P. Polymorphisms in genes expressed during amelogenesis and their association with dental caries: a case-control study. *Clin. Oral Invest*. **2023**, *27*, 1681–1695. https://doi.org/10.1007/S00784-022-04794-2

17. Gerreth, K., Zaorska, K., Zabel, M., Borysewicz-Lewicka, M., Nowicki, M. Association of ENAM gene single nucleotide polymorphisms with dental caries in Polish children. *Clin. Oral Invest*. **2016**, *20*, 631. https://doi.org/10.1007/S00784-016-1743-1
18. Gerreth, K., Zaorska, K., Zabel, M., Borysewicz-Lewicka, M., Nowicki, M. Chosen single nucleotide polymorphisms (SNPs) of enamel formation genes and dental caries in a population of Polish children. *Adv. Clin Exp Med*. **2017**, *26*, 899–905. https://doi.org/10.17219/ACEM/63024
19. Haznedaroɫlu, E., Koldemir-Gündüz, M., Bakr-Coşkun, N., Bozkuş, H.M., Çaɫatay, P., Süsleyici-Duman, B., Menteş, A. Association of sweet taste receptor gene polymorphisms with dental caries experience in school children. *Caries Res*. **2015**, *49*, 275–281. https://doi.org/10.1159/000381426
20. Holla, L.I., Borilova Linhartova, P., Lucanova, S., Kastovsky, J., Musilova, K., Bartosova, M., Kukletova, M., Kukla, L., Dusek, L. GLUT2 and TAS1R2 Polymorphisms and Susceptibility to Dental Caries. *Caries Res*. **2015**, *49*, 417–424. https://doi.org/10.1159/000430958
21. Hu, X.P., Song, T.Z., Zhu, Y.Y., Wu, L.L., Zhang, X., Zhou, J.Y., Li, Z.Q. Association of ENAM, TUFT1, MMP13, IL1B, IL10 and IL1RN gene polymorphism and dental caries susceptibility in Chinese children. *J. Int. Med. Res*. **2019**, *47*, 1696–1704. https://doi.org/10.1177/0300060519828450
22. Hu, X.P., Zhou, H.J., Li, Z.Q., Song, T.Z., Zhu, Y.Y. Lack of associations between lactoferrin (LTF) and mannose-binding lectin 2 (MBL2) gene polymorphism and dental caries susceptibility. *J. Int. Med. Res*. **2020**, *48*. https://doi.org/10.1177/0300060520943428
23. Izakovicova Holla, L., Borilova Linhartova, P., Kastovsky, J., Bartosova, M., Musilova, K., Kukla, L., Kukletova, M. Vitamin D Receptor TaqI Gene Polymorphism and Dental Caries in Czech Children. *Caries Res*. **2017**, *51*, 7–11. https://doi.org/10.1159/000452635
24. Jeremias, F., Koruyucu, M., Küchler, E.C., Bayram, M., Tuna, E.B., Deeley, K., Pierri, R.A., Souza, J.F., Fragelli, C.M.B., Paschoal, M.A.B., Gencay, K., Seymen, F., Caminaga, R.M.S., Dos Santos-Pinto, L., Vieira, A.R. Genes expressed in dental enamel development are associated with molar-incisor hypomineralization. *Arch. Oral Biol*. **2013**, *58*, 1434–1442. https://doi.org/10.1016/j.archoralbio.2013.05.005
25. Kang, S., Yoon, I., Lee, H., Cho, J. Association between AMELX polymorphisms and dental caries in Koreans. *Oral Dis*. **2011**, *17*, 399–406. https://doi.org/10.1111/J.1601-0825.2010.01766.X
26. Kelly, A.M., Bezamat, M., Modesto, A., Vieira, A.R. Biomarkers for Lifetime Caries-Free Status. *J. Pers. Med*. **2021**, *11*, 23. https://doi.org/10.3390/JPM11010023
27. Kong, Y-Y., Zheng, J-M., Zhang, W-Y., Jiang, Q-Z., Yang, X-C., Yu, M., Zeng, S-J. The relationship between vitamin D receptor gene polymorphism and deciduous tooth decay in Chinese children. *BMC Oral Health.* 2017, *17*, 111. https://doi.org/10.1186/S12903-017-0398-X
28. Li, X., Liu, D., Sun, Y., Yang, J.,Yu, Y. Association of genetic variants in enamel-formation genes with dental caries: A meta- and gene-cluster analysis. *Saudi J. Biol. Sci*. **2021**, *28*, 1645–1653. https://doi.org/10.1016/J.SJBS.2020.11.071
29. Liang, Y., Yao, J., Qiu, R., Chen, A., Huang, H., Lin, H., Yu, L. The rs35874116 single nucleotide polymorphism increases sweet intake and the risk of severe early childhood caries: a case–control study. *BMC Oral Health.* **2022**, *22*, 471. https://doi.org/10.1186/S12903-022-02512-8
30. Mossad, H., Negm, H. Do Enamelin, Lactotransferrin, and Amelogenin-X Polymorphisms Predispose Dental Caries Susceptibility in Egyptian Children? A Cross-Sectional Study. *Journal of the California Dental Association*. **2023**, *51*. https://doi.org/10.1080/19424396.2023.2237216
31. Negm, H.M.H., Farag, A.F., Taha, R.R.O.O. Polymorphisms in ENAM, AMBN, and KLK4 predispose Egyptian adults to dental caries: A cross-sectional study. *The Saudi Dental Journal*. **2024**, *36*, 915. https://doi.org/10.1016/J.SDENTJ.2024.03.014
32. Pang, L., Wang, K., Tao, Y., Zhi, Q., Zhang, J., Lin, H. A New Model for Caries Risk Prediction in Teenagers Using a Machine Learning Algorithm Based on Environmental and Genetic Factors. *Front. Genet*. **2021**, *12*. https://doi.org/10.3389/FGENE.2021.636867
33. Pehlivan, S., Koturoglu, G., Ozkinay, F., Alpoz, A.R., Sipahi, M., Pehlivan, M. Might there be a link between mannose-binding lectin polymorphism and dental caries? *Mol. Immunol*. **2005**, *42*, 1125–1127. https://doi.org/10.1016/J.MOLIMM.2004.10.002

34. Qin, X., Shao, L., Zhang, L., Ma, L.,Xiong, S. Investigation of Interaction between Vitamin D Receptor Gene Polymorphisms and Environmental Factors in Early Childhood Caries in Chinese Children. *BioMed Res Int*. **2019**. https://doi.org/10.1155/2019/4315839
35. Reza Khami, M., Asgari, S., Valizadeh, S., Karami, J., Rezaei, A., Rezaei, N. AMELX and ENAM Polymorphisms and Dental Caries. *Int. J. Dent*. **2022**. https://doi.org/10.1155/2022/8501179
36. Shimomura-Kuroki, J., Nashida, T., Miyagawa, Y., Sekimoto, T. The Role of Genetic Factors in the Outbreak Mechanism of Dental Caries. *The Journal of Clinical Pediatric Dentistry.* **2018**, *42*, 32–36. https://doi.org/10.17796/1053-4628-42.1.6
37. Tannure, P.N., Küchler, E.C., Falagan-Lotsch, P., Amorim, L.M.F., Raggio Luiz, R., Costa, M.C., Vieira, A.R., Granjeiro, J.M. MMP13 polymorphism decreases risk for dental caries. *Caries Res*. **2012**, *46*, 401–407. https://doi.org/10.1159/000339379
38. Tannure, P.N., Küchler, E.C., Lips, A., Costa, M.D.C., Luiz, R.R., Granjeiro, J.M., Vieira, A.R. Genetic variation in MMP20 contributes to higher caries experience. *J. Dent*. **2012**, *40*, 381–386. https://doi.org/10.1016/J.JDENT.2012.01.015
39. Vasconcelos, K.R., Arid, J., Evangelista, S., Oliveira, S., Dutra, A.L., Silva, L.A.B., Segato, R.A.B., Vieira, A.R., Nelson-Filho, P., Küchler, E.C. MMP13 Contributes to Dental Caries Associated with Developmental Defects of Enamel. *Caries Res*. **2019**, *53*, 441–446. https://doi.org/10.1159/000496372
40. Volckova, M., Borilova Linhartova, P., Trefna, T., Vlazny, J., Musilova, K., Kukletova, M., Kukla, L., Izakovicova Holla, L. Lack of association between lactotransferrin polymorphism and dental caries. *Caries Res*. **2014**, *48*, 39–44. https://doi.org/10.1159/000351689
41. Wang, M., Qin, M. Lack of association between LTF gene polymorphisms and different caries status in primary dentition. *Oral Dis*. **2018**, *24*, 1545–1553. https://doi.org/10.1111/ODI.12939
42. Wang, M., Qin, M., Xia, B. The association of Enamelin, Lactoferrin, and Tumour necrosis factor alpha gene polymorphisms with high caries susceptibility in Chinese children under 4 years old. *Arch. Oral Biol*. **2017**, *80*, 75–81. https://doi.org/10.1016/j.archoralbio.2017.03.023
43. Yang, Y., Wang, W., Qin, M. Mannose-binding lectin gene polymorphisms are not associated with susceptibility to severe early childhood caries. *Hum. Immunol*. **2013**, *74*, 110–113. https://doi.org/10.1016/J.HUMIMM.2012.08.012
44. Yildiz, G., Ermis, R.B., Calapoglu, N.S., Celik, E.U., Türel, G.Y. Gene-environment Interactions in the Etiology of Dental Caries. *J. Dent. Res*. **2016**, *95*, 74–79. https://doi.org/10.1177/0022034515605281
45. Yilmaz, M., Balci, S., Topbas, N.K., Yildirim, D.D., Tamer, L. Association of bitter and sweet taste gene receptor polymorphisms with dental caries formation. *Turk. J. Biochem*. **2021**, *46*, 721–727. https://doi.org/10.1515/TJB-2019-0153
46. Yu, M., Jiang, Q.Z., Sun, Z.Y., Kong, Y.Y., Chen, Z. Association between Single Nucleotide Polymorphisms in Vitamin D Receptor Gene Polymorphisms and Permanent Tooth Caries Susceptibility to Permanent Tooth Caries in Chinese Adolescent. *BioMed Res. Int*. **2017**, 4096316. https://doi.org/10.1155/2017/4096316
47. Zaorska, K., Szczapa, T., Borysewicz-Lewicka, M., Nowicki, M., Gerreth, K. Prediction of Early Childhood Caries Based on Single Nucleotide Polymorphisms Using Neural Networks. *Genes*. **2021**, *12*. https://doi.org/10.3390/GENES12040462
48. Sharifi, R.; Jahedi, S.; Mozaffari, H.R.; Imani, M.M.; Sadeghi, M.; Golshah, A.; Moradpoor, H.; Safaei, M. Association of Ltf, Enam, and Amelx Polymorphisms with Dental Caries Susceptibility: A Meta-Analysis. *BMC Oral Health* **2020**, *20*, 132.
49. Chisini, L.A.; de Carvalho, R.V.; Santos Costa, F.D.; Salvi, L.C.; Demarco, F.F.; Correa, M.B. Genes and Single Nucleotide Polymorphisms in the Pathway of Saliva and Dental Caries: A Systematic Review and Meta-Analysis. *Biofouling* **2023**, *39*, 8–23.
50. Chisini, L.A.; Salvi, L.C.; de Carvalho, R.V.; Santos Costa, F.d.; Demarco, F.F.; Correa, M.B. Pathways of the Vitamin D Receptor Gene and Dental Caries: A Systematic Review and Meta-Analysis. *Arch. Oral Biol.* **2025**, *173*, 106195.
51. Lips, A.; Antunes, L.S.; Antunes, L.A.; Pintor, A.V.B.; Santos, D.A.B.; Bachinski, R.; Küchler, E.C.; Alves, G.G. Salivary Protein Polymorphisms and Risk of Dental Caries: A Systematic Review. *Braz. Oral Res.* **2017**, *31*, e41.
52. Oliveira, D.S.B.; Segato, R.A.B.; Oliveira, S.; Dutra, A.L.T.; Santos, A.S.; Praxedes, A.N.; Belém, L.C.; Antunes, L.A.; Lips, A.; Nelson-Filho, P.; da Silva, L.A.B.; Alves, G.G.; Antunes, L.S.; Küchler, E.C. Association between

genetic polymorphisms in DEFB1 and microRNA202 with caries in two groups of Brazilian children. *Arch. Oral Biol.* **2018**, *92*, 1–7. https://doi.org/10.1016/j.archoralbio.2018.04.010.
53. Koohpeima, F.; Hashemi-Gorji, F.; Mokhtari, M.J. Evaluation of caries experience in two genders and ENAM polymorphism in Iranian adults. *Meta Gene* **2018**, *17*, 78–81.
54. Qin, X.; Wang, M.; Wang, L.; Xu, Y.; Xiong, S. Association of Vitamin D Receptor Gene Polymorphisms with Caries Risk in Children: A Systematic Review and Meta-Analysis. *BMC Pediatr.* **2024**, *24*, 650.
55. Chisini, L.A.; Cademartori, M.G.; Conde, M.C.M.; Santos Costa, F.D.; Tovo-Rodrigues, L.; de Carvalho, R.V.; Demarco, F.F.; Correa, M.B. Genes and Snps in the Pathway of Immune Response and Caries Risk: A Systematic Review and Meta-Analysis. *Biofouling* **2020**, *36*, 1100–1116.
56. Chisini, L.A.; Cademartori, M.G.; Conde, M.C.M.; Costa, F.d.S.; Salvi, L.C.; Tovo-Rodrigues, L.; Correa, M.B. Single Nucleotide Polymorphisms of Taste Genes and Caries: A Systematic Review and Meta-Analysis. *Acta Odontol. Scand.* **2021**, *79*, 147–155.
57. Chisini, L.A.; Cademartori, M.G.; Muniz Conde, M.C.; Tovo-Rodrigues, L.; Correa, M.B. Genes in the Pathway of Tooth Mineral Tissues and Dental Caries Risk: A Systematic Review and Meta-Analysis. *Clin. Oral Investig.* **2020**, *24*, 3723–3738.
