# Supplementary material for: Genetic Variants and Dental Caries Susceptibility: An Umbrella Review and Multilevel Meta-Analysis
Source: Genes (Basel). 2026 Jun 22;17(6):724. doi: 10.3390/genes17060724 (PMC13299435; doi:10.3390/genes17060724)
Supplement: Supplementary file 1 [file genes-17-00724-s001.zip › Supplementary Table S2.pdf]

Supplementary Table S2. Jadad-Based Review Selection

Category	Gene	SNP (rsID)	Study	Jadad/selected SR
Immune and inflammatory responses	LTF	rs1126478	Shojaei et al., 2026	Shojaei et al., 2026 [1]
			Aruna et al., 2023	
			Sharifi et al., 2020	
			Li et al., 2020	
			Chisini et al., 2020a	
		rs11216477	Li et al., 2020	Li et al., 2020 [2]
		rs6441989	Li et al., 2020	
			Chisini et al., 2020a	Chisini et al., 2020a [3]
		rs2073495	Li et al., 2020	
			Chisini et al., 2020a	Chisini et al., 2020a [3]
		rs11716497	Li et al., 2020	Li et al., 2020 [2]
		rs1126477	Aruna et al., 2023	
			Chisini et al., 2020a	Chisini et al., 2020a [3]
		rs2269436	Aruna et al., 2023	
			Chisini et al., 2020a	Chisini et al., 2020a [3]
		rs743658	Aruna et al., 2023	
			Chisini et al., 2020a	Chisini et al., 2020a [3]
		rs4547741	Aruna et al., 2023	
			Chisini et al., 2020a	Chisini et al., 2020a [3]
		rs1126478	Aruna et al., 2023	Aruna et al., 2023 [4]
		rs17078878	Aruna et al., 2023	Aruna et al., 2023 [4]
	ALOX15	rs2619112	Aruna et al., 2023	Aruna et al., 2023 [4]
		rs7217186	Aruna et al., 2023	Aruna et al., 2023 [4]
	DEFB1	rs11362	HatipogluO et al., 2020	
			Ślebioda et al., 2021	Ślebioda et al., 2021 [5]
			Hemati et al., 2023	
			Aruna et al., 2023	
			Chisini et al., 2020a	Chisini et al., 2020a [3]
		rs1799946	Ślebioda et al., 2021	
			Hemati et al., 2023	Hemati et al., 2023 [6]
			Aruna et al., 2023	
			Chisini et al., 2020a	
		rs1047031	Ślebioda et al., 2021	
		rs1800972	Ślebioda et al., 2021	Ślebioda et al., 2021 [5]
			Hemati et al., 2023	Hemati et al., 2023 [6]
			Aruna et al., 2023	
			Chisini et al., 2020a	
	MASP2	rs72550870	Chisini et al., 2020a	Chisini et al., 2020a [3]
			Aruna et al., 2023	
		rs2672812	Chisini et al., 2020a	
		rs2672785	Chisini et al., 2020a	
	TNF-α	rs1800629	Aruna et al., 2023	
	MBL2	rs7096206	Hemati et al., 2023	Hemati et al., 2023 [6]
			Chisini et al., 2020a	Chisini et al., 2020a [3]
			Aruna et al., 2023	

		rs1800450	Hemati et al., 2023	
			Chisini et al., 2020a	Chisini et al., 2020a [3]
			Aruna et al., 2023	
		rs11003125	Hemati et al., 2023	
			Chisini et al., 2020a	
			Aruna et al., 2023	
	TRAV4	rs1997532	Aruna et al., 2023	
		rs8011979	Aruna et al., 2023	
		rs7150049	Aruna et al., 2023	
		rs1997533	Aruna et al., 2023	
	TIMP1	rs4898	Chisini et al., 2020	Chisini et al., 2020a [3]
			Sharma et al., 2023	
	TIMP2	rs7501477	Chisini et al., 2020	
	LPO	rs8178350	Aruna et al., 2023	Aruna et al., 2023 [4]
		rs7209537	Aruna et al., 2023	
		rs17762644	Aruna et al., 2023	
		rs8178281	Aruna et al., 2023	
		rs8178290	Aruna et al., 2023	
		rs8178307	Aruna et al., 2023	
		rs8178329	Aruna et al., 2023	
		rs3744093	Aruna et al., 2023	
Vitamin D receptor	Bsml	rs1544410	Chisin et al., 2025	
			Qin et al., 2024	Qin et al., 2024 [7]
			Sadeghi et al., 2021	
	ApaI	rs7975232	Chisin et al., 2025	Chisin et al., 2025 [8]
			Qin et al., 2024	
			Sadeghi et al., 2021	
	FokI	rs10735810	Chisin et al., 2025	Chisin et al., 2025 [8]
			Qin et al., 2024	
			Sadeghi et al., 2021	
	Cdx2	rs11568820	Chisin et al., 2025	
	3’ UTR var	rs739837	Chisin et al., 2025	
			Qin et al., 2024	Qin et al., 2024 [7]
			Sadeghi et al., 2021	
	FokI (alt ID)	rs2228570	Chisin et al., 2025	
			Qin et al., 2024	Qin et al., 2024 [7]
			Sadeghi et al., 2021	
	TaqI	rs731236	Sadeghi et al., 2021	
			Lei et al., 2021	Lei et al., 2021 [9]
			Qin et al., 2024	
			Chisin et al., 2025	Chisin et al., 2025 [8]
Tooth development and mineralization	AMBN	rs4694075	Sharifi et al., 2021	
			Sharma et al., 2023	
			Chisini et al., 2020b	Chisini et al., 2020b [10]
		rs34538475	Sharifi et al., 2021	
			Sharma et al., 2023	
			Chisini et al., 2020b	Chisini et al., 2020b [10]
		rs7439186	Sharma et al., 2023	
		rs3124953	Chisini et al., 2020b	
	ENAM	rs1260848	Chisini et al., 2020b	

		rs3796703	Shojaei et al., 2026	
		rs3796703	Sharifi et al., 2020	
			Chisini et al., 2020b	
		rs1264848	Sharifi et al., 2020	
		rs3796704	Sharifi et al., 2020	
			Li, X et al., 2021	Li et al., 2021 [15]
			Chisini et al., 2020b	
		rs12640848	Li, X et al., 2021	Li et al., 2021 [15]
			Chisini et al., 2020b	
	TUFT1	rs4970957	Sharifi et al., 2021	
			Sharma et al., 2023	
			Chisini et al., 2020b	Chisini et al., 2020b [10]
		rs10158855	Sharma et al., 2023	
		rs2337359	Sharma et al., 2023	
		rs12749	Sharma et al., 2023	
		rs2337359	Sharma et al., 2023	
	AMELX	rs6639060	Sharifi et al., 2020	
			Chisini et al., 2020b	
		rs2106416	Sharifi et al., 2020	Sharifi et al., 2020 [14]
			Chisini et al., 2020b	
		rs5933871	Chisini et al., 2020b	
		rs5934997	Chisini et al., 2020b	
		rs7052450	Chisini et al., 2020b	
		rs946252	Sharifi et al., 2020	
			Chisini et al., 2020b	
		rs17878486	Sharifi et al., 2020	Sharifi et al., 2020 [14]
			Li, X et al., 2021	
			Shojaei et al., 2026	Shojaei et al., 2026 [16]
			Chisini et al., 2020b	
	TFIP11	rs5997096	Chisini et al., 2020b	Chisini et al., 2020b [10]
		rs134136	Chisini et al., 2020b	
		rs2609428	Chisini et al., 2020b	
	KLK4	rs2235091	Chisini et al., 2020b	
			Li et al., 2023	
			Sharma et al., 2023	Sharma et al., 2023 [11]
		rs198968	Chisini et al., 2020b	
			Sharma et al., 2023	
		rs198969	Chisini et al., 2020b	
			Sharma et al., 2023	Sharma et al., 2023 [11]
		rs198966	Sharma et al., 2023	
		rs2242670	Chisini et al., 2020b	
		rs2978642	Chisini et al., 2020b	
		rs2978643	Chisini et al., 2020b	Chisini et al., 2020b [10]
	MMP9	rs3790506	Sharifi et al., 2021	Sharifi et al., 2021 [12]
			Chisini et al., 2020b	
		rs3828054	Chisini et al., 2020b	
		rs17576	Molaei et al., 2022	
			Sharma et al., 2023	
			Chisini et al., 2020b	
	MMP13	rs2252070	Molaei et al., 2022	

			Najafi-Ghobadi et al., 2023	Najafi-Ghobadi et al., 2023 [13]
			Sharma et al., 2023	
			Chisini et al., 2020b	
	MMP16	rs17719876	Sharma et al., 2023	Sharma et al., 2023 [11]
		rs17720688	Sharma et al., 2023	
		rs16878625	Sharma et al., 2023	
		rs6469206	Sharma et al., 2023	
		rs1824717	Sharma et al., 2023	
		rs7826929	Sharma et al., 2023	
		rs10103111	Sharma et al., 2023	
		rs2616487	Sharma et al., 2023	
		rs10089111	Sharma et al., 2023	
		rs17718917	Sharma et al., 2023	
		rs1382104	Sharma et al., 2023	
		rs1551893	Sharma et al., 2023	
		rs2054415	Sharma et al., 2023	
		rs1477907	Sharma et al., 2023	
		rs16876790	Sharma et al., 2023	
		rs2046315	Sharma et al., 2023	
	MMP20	rs1784418	Molaei et al., 2022	
			Li et al., 2021	
			Chisini et al., 2020b	
		rs1711437	Chisini et al., 2020b	
			Sharma et al., 2023	
	MMP	rs17514136	Chisini et al., 2020b	
	MMP2	rs243847	Chisini et al., 2020b	
		rs243865	Chisini et al., 2020b	
			Sharma et al., 2023	
	MMP3	rs522616	Chisini et al., 2020b	
	BMP2	rs1884302	Chisini et al., 2020b	
	BMP4	rs2761887	Chisini et al., 2020b	
	BMP7	rs388286	Chisini et al., 2020b	
	AMBN	rs3924573	Sharma et al., 2023	
	TUFT1	rs2337360	Sharma et al., 2023	Sharma et al., 2023 [11]
			Chisini et al., 2020b	Chisini et al., 2020b [10]
		rs1045298	Sharma et al., 2023	
		rs7526319	Sharma et al., 2023	
			Chisini et al., 2020b	
		rs8934	Sharma et al., 2023	
	DLX3	rs10459948	Chisini et al., 2020b	Chisini et al., 2020b [10]
		rs11656951	Chisini et al., 2020b	
		rs12452477	Chisini et al., 2020b	
		rs16948563	Chisini et al., 2020b	
		rs2278163	Chisini et al., 2020b	
		rs2303466	Chisini et al., 2020b	
		rs3891034	Chisini et al., 2020b	
Taste perception and signaling	TAS1R2	rs35874116	Motahari et al., 2024	
			Chisini et al., 2021	
		rs9701796	Motahari et al., 2024	

			Chisini et al., 2021	
		rs3935570	Chisini et al., 2021	
		rs4920566	Chisini et al., 2021	
	TAS2R38	rs713598	Chisini et al., 2021	Chisini et al., 2021 [18]
		rs1726866	Chisini et al., 2021	
		rs10246939	Chisini et al., 2021	
	TAS1R3	rs307355	Chisini et al., 2021	Chisini et al., 2021 [18]
		rs1499821	Chisini et al., 2021	
	GLUT2	rs5398	Chisini et al., 2021	Chisini et al., 2021 [18]
		rs5400	Chisini et al., 2021	
		rs11924032	Chisini et al., 2021	
Salivary composition and function	CA6	rs2274328	Chisini et al., 2023	Chisini et al., 2023 [17]
			Sharifi et al., 2021	
			Hatipoglu et al., 2019	
		rs2274333	Chisini et al., 2023	Chisini et al., 2023 [17]
			Sharifi et al., 2021	
			Hatipoglu et al., 2019	
		rs2274327	Chisini et al., 2023	Chisini et al., 2023 [17]
			Sharifi et al., 2021	
			Hatipoglu et al., 2019	
	AQP5	rs10875989	Chisini et al., 2023	Chisini et al., 2023 [17]
		rs17032907	Chisini et al., 2023	
		rs467323	Chisini et al., 2023	
		rs923911	Chisini et al., 2023	
		rs3759129	Chisini et al., 2023	
		rs1996315	Chisini et al., 2023	
	AQP2	rs10864376	Chisini et al., 2023	
		rs12021597	Chisini et al., 2023	
		rs12138897	Chisini et al., 2023	
	MUC5B	rs2735733	Chisini et al., 2023	
			Chisini et al., 2020a	
		rs2249073	Chisini et al., 2023	
			Chisini et al., 2020a	
		rs2857476	Chisini et al., 2023	
			Chisini et al., 2020a	

References:

1. Shojaei, D.; Sadat Mohammadipour, H.; Sekandari, S.; Dehghani, M.; Mohajertehran, F. The Role of Ltf, Enam, and Amelx Gene Polymorphisms in Dental Caries Susceptibility: A Meta-Analysis. *Curr. Genet. Med. Rep.* **2026**, *14*, 4.
2. Li, X.; Su, Y.; Liu, D.; Yang, J. The Association between Genetic Variants in Lactotransferrin and Dental Caries: A Meta- and Gene-Based Analysis. *BMC Med. Genet* **2020**, *21*, 114.
3. Chisini, L.A.; Cademartori, M.G.; Conde, M.C.M.; Santos Costa, F.D.; Tovo-Rodrigues, L.; de Carvalho, R.V.; Demarco, F.F.; Correa, M.B. Genes and Snps in the Pathway of Immune Response and Caries Risk: A Systematic Review and Meta-Analysis. *Biofouling* **2020**, *36*, 1100–1116.

4. Aruna, P.; Patil, S.S.; Muthu, M.S.; Vettriselvi, V.; Arockiam, S.; Kirubakaran, R.; Sivakumar, N. Association between Polymorphisms of Immune Response Genes and Early Childhood Caries—Systematic Review, Gene-Based, Gene Cluster, and Meta-Analysis. *J. Genet Eng. Biotechnol.* **2023**, *21*, 124.
5. Ślebioda, Z.; Woźniak, T.; Dorocka-Bobkowska, B.; Woźniewicz, M.; Kowalska, A. Beta-Defensin 1 Gene Polymorphisms in the Pathologies of the Oral Cavity-Data from Meta-Analysis: Association Only with Rs1047031 Not with Rs1800972, Rs1799946, and Rs11362. *J. Oral Pathol. Med.* **2021**, *50*, 22–31.
6. Hemati, G.; Imani, M.M.; Choubsaz, P.; Inchingolo, F.; Sharifi, R.; Sadeghi, M.; Tadakamadla, S.K. Evaluation of Beta-Defensin 1 and Mannose-Binding Lectin 2 Polymorphisms in Children with Dental Caries Compared to Caries-Free Controls: A Systematic Review and Meta-Analysis. *Children* **2023**, *10*, 232
7. Qin, X.; Wang, M.; Wang, L.; Xu, Y.; Xiong, S. Association of Vitamin D Receptor Gene Polymorphisms with Caries Risk in Children: A Systematic Review and Meta-Analysis. *BMC Pediatr.* **2024**, *24*, 650.
8. Chisini, L.A.; Salvi, L.C.; de Carvalho, R.V.; Santos Costa, F.d.; Demarco, F.F.; Correa, M.B. Pathways of the Vitamin D Receptor Gene and Dental Caries: A Systematic Review and Meta-Analysis. *Arch. Oral Biol.* **2025**, *173*, 106195.
9. Lei, W.; Tian, H.; Xia, Y. Association between the Taqi (Rs731236 T>C) Gene Polymorphism and Dental Caries Risk: A Meta-Analysis. *Genet Test. Mol. Biomark.* **2021**, *25*, 368–375.
10. Chisini, L.A.; Cademartori, M.G.; Muniz Conde, M.C.; Tovo-Rodrigues, L.; Correa, M.B. Genes in the Pathway of Tooth Mineral Tissues and Dental Caries Risk: A Systematic Review and Meta-Analysis. *Clin. Oral Investig.* **2020**, *24*, 3723–3738.
11. Sharma, A.; Patil, S.S.; Muthu, M.S.; Venkatesan, V.; Kirubakaran, R.; Nuvvula, S.; Arockiam, S. Single Nucleotide Polymorphisms of Enamel Formation Genes and Early Childhood Caries—Systematic Review, Gene-Based, Gene Cluster and Meta-Analysis. *J. Indian Soc. Pedod. Prev. Dent.* **2023**, *41*, 3–15.
12. Sharifi, R.; Shayan, A.; Jamshidy, L.; Mozaffari, H.R.; Hatipoğlu, Ö.; Tadakamadla, S.K.; Sadeghi, M. A Systematic Review and Meta-Analysis of Ca Vi, Ambn, and Tuft1 Polymorphisms and Dental Caries Risk. *Meta Gene* **2021**, *28*, 100866.
13. Najafi-Ghobadi, K.; Rajabi-Moghaddam, M.; Abbaszadeh, H. The Association between Mmp13 Rs2252070 Polymorphism and Caries Susceptibility: A Systematic Review and Meta-Analysis. *Hum. Gene* **2023**, *35*, 201143.
14. Sharifi, R.; Jahedi, S.; Mozaffari, H.R.; Imani, M.M.; Sadeghi, M.; Golshah, A.; Moradpoor, H.; Safaei, M. Association of Ltf, Enam, and Amelx Polymorphisms with Dental Caries Susceptibility: A Meta-Analysis. *BMC Oral Health* **2020**, *20*, 132.
15. Li, X.; Liu, D.; Sun, Y.; Yang, J.; Yu, Y. Association of Genetic Variants in Enamel-Formation Genes with Dental Caries: A Meta- and Gene-Cluster Analysis. *Saudi J. Biol. Sci.* **2021**, *28*, 1645–1653.
16. Shojaei, D.; Sadat Mohammadipour, H.; Sekandari, S.; Dehghani, M.; Mohajertehran, F. The Role of Ltf, Enam, and Amelx Gene Polymorphisms in Dental Caries Susceptibility: A Meta-Analysis. *Curr. Genet. Med. Rep.* **2026**, *14*, 4.
17. Chisini, L.A.; de Carvalho, R.V.; Santos Costa, F.D.; Salvi, L.C.; Demarco, F.F.; Correa, M.B. Genes and Single Nucleotide Polymorphisms in the Pathway of Saliva and Dental Caries: A Systematic Review and Meta-Analysis. *Biofouling* **2023**, *39*, 8–23.
18. Chisini, L.A.; Cademartori, M.G.; Conde, M.C.M.; Costa, F.d.S.; Salvi, L.C.; Tovo-Rodrigues, L.; Correa, M.B. Single Nucleotide Polymorphisms of Taste Genes and Caries: A Systematic Review and Meta-Analysis. *Acta Odontol. Scand.* **2021**, *79*, 147–155.
