# Supplementary material for: Genetic Variants and Dental Caries Susceptibility: An Umbrella Review and Multilevel Meta-Analysis
Source: Genes (Basel). 2026 Jun 22;17(6):724. doi: 10.3390/genes17060724 (PMC13299435; doi:10.3390/genes17060724)
Supplement: Supplementary file 1 [file genes-17-00724-s001.zip › Supplementary Table S3.pdf]

Supplementary Table S3. Excluded studies and reasons for exclusion

Title	Author	Exclusion reason
Polymorphisms associated with dental caries in pediatric populations: a systematic review	González-Casamada et al., 2023 [1]	The full text is not in English
Is there an association between dental caries and genetics? Systematic review and meta-analysis of studies with twins.	DosAnjos et al., 2023 [2]	Wrong intervention
Genome-wide association meta-analysis identifies two novel loci associated with dental caries.	Nogawa et al., 2024 [3]	Wrong study design
A Polygenic Score Predicts Caries Experience in Elderly Swedish Adults.	Fries et al., 2024 [4]	Wrong study design
Gene sequencing applications to combat oral-cavity related disorders: a systematic review with meta-analysis.	Abdul et al., 2024 [5]	Wrong outcomes
Association of Early Childhood Caries with Bitter Taste Receptors: A Meta-Analysis of Genome-Wide Association Studies and Transcriptome-Wide Association Study.	Orlova et al., 2022 [6]	Wrong study design
Genome-wide analysis of dental caries and periodontitis combining clinical and self-reported data.	Shungin et al., 2019 [7]	Wrong study design
Protocols, Methods, and Tools for Genome-Wide Association Studies (GWAS) of Dental Traits.	Agler et al., 2019 [8]	Wrong study design
Consortium-based genome-wide meta-analysis for childhood dental caries traits.	Haworth et al., 2018 [9]	Wrong study design
Genetic Association of MMP10, MMP14, and MMP16 with Dental Caries.	Lewis et al., 2017 [10]	Wrong study design
Interaction of lifestyle, behaviour or systemic diseases with dental caries and periodontal diseases: consensus report of group 2 of the joint EFP/ORCA workshop on the boundaries between caries and periodontal diseases.	Chapple et al.,2017 [11]	Wrong study design
Variants on chromosome 4q21 near PKD2 and SIBLINGs are associated with dental caries.	Eckert et al., 2017 [12]	Wrong study design
MMP20 rs1784418 Protects Certain Populations against Caries.	Filho et al., 2017 [13]	Wrong study design
Host genetics role in the pathogenesis of periodontal disease and caries.	Nibali et al., 2017 [14]	Wrong exposure and outcomes
Meta-Analysis of Genome-Wide Association Studies with Correlated Individuals: Application to the Hispanic Community Health Study/Study of Latinos (HCHS/SOL).	Sofer et al., 2016 [15]	Wrong study design
Genome-wide association study of dental caries in the Hispanic Communities Health Study/Study of Latinos (HCHS/SOL).	Morrison et al., 2016 [16]	Wrong study design
Effects of enamel matrix genes on dental caries are moderated by fluoride exposures.	Shaffer et al., 2015 [17]	Wrong study design
Genetic Association of MPPED2 and ACTN2 with Dental Caries.	Stanley et al., 2014 [18]	Wrong study design
Genome-wide association scan of dental caries in the permanent dentition.	Wang et al., 2012 [19]	Wrong study design
Inherited risks for susceptibility to dental caries.	Shuler et al., 2001 [20]	Wrong study design
Correlation between hla-dqb1 and hla-drb1 gene polymorphisms and caries: A systematic review and meta-analysis	Chen et al., 2021 [21]	The full text is not in English

Genetic Risk Score to Predict the Likelihood of Dental Caries in Finnish Adolescents	Agrawal et al., 2021 [22]	Wrong study design
The dessert dimension: Genes related to sweet taste receptors, sweet taste preferences, its association with alcohol consumption and dental caries incidence – a literature synthesis and scoping review	Kumar et al., 2021 [23]	Wrong study design
State of dentition among twins considering the influence of genetic and environmental factors: The systematic review of the literature	Piekoszewska-Ziętek et al., 2016 [24]	Wrong intervention
The Role of Genetic on the Risk for Caries in Primary Teeth: A Scoping Review	Yulianti et al., 2023 [25]	Wrong study design

References:

1. González-Casamada, C.; Molina-Frechero, N.; Espinosa-Cristóbal, L.F.; García-López, S.; Castañeda-Castaneira, E. Polymorphisms Associated with Dental Caries in Pediatric Populations: A Systematic Review. *Rev. Med. Inst. Mex. Seguro Soc.* **2023**, *61*, 502–508. https://doi.org/10.5281/zenodo.8200501
2. Dos Anjos, A.M.C.; Moura de Lima, M.D.; Muniz, F.W.M.G.; Lima, C.C.B.; Moura, L.F.A.D.; Rösing, C.K.; de Moura, M.S. Is There an Association between Dental Caries and Genetics? Systematic Review and Meta-Analysis of Studies with Twins. *J. Dent.* **2023**, *135*, 104586. https://doi.org/10.1016/j.jdent.2023.104586
3. Nogawa, S.; Morishita, S.; Saito, K.; Kato, H. Genome-Wide Association Meta-Analysis Identifies Two Novel Loci Associated with Dental Caries. *BMC Oral Health* **2024**, *24*, 1003. https://doi.org/10.1186/s12903-024-04799-1
4. Fries, N.; Haworth, S.; Shaffer, J.R.; Esberg, A.; Divaris, K.; Marazita, M.L.; Johansson, I. A Polygenic Score Predicts Caries Experience in Elderly Swedish Adults. *J. Dent. Res.* **2024**, *103*, 502–508. https://doi.org/10.1177/00220345241232330
5. Abdul, N.S.; Shenoy, M.; Reddy, N.R.; Sangappa, S.B.; Shivakumar, G.C.; Di Blasio, M.; Cicciù, M.; Minervini, G. Gene Sequencing Applications to Combat Oral-Cavity Related Disorders: A Systematic Review with Meta-Analysis. *BMC Oral Health* **2024**, *24*, 103. https://doi.org/10.1186/s12903-023-03541-7
6. Orlova, E.; Dudding, T.; Chernus, J.M.; Alotaibi, R.N.; Haworth, S.; Crout, R.J.; Lee, M.K.; Mukhopadhyay, N.; Feingold, E.; Levy, S.M.; et al. Association of Early Childhood Caries with Bitter Taste Receptors: A Meta-Analysis of Genome-Wide Association Studies and Transcriptome-Wide Association Study. *Genes* **2023**, *14*, 59. https://doi.org/10.3390/genes14010059
7. Shungin, D.; Haworth, S.; Divaris, K.; Agler, C.S.; Kamatani, Y.; Lee, M.K.; Grinde, K.; Hindy, G.; Alaraudanjoki, V.; Pesonen, P.; et al. Genome-Wide Analysis of Dental Caries and Periodontitis Combining Clinical and Self-Reported Data. *Nat. Commun.* **2019**, *10*, 2773. https://doi.org/10.1038/s41467-019-10630-1
8. Agler, C.S.; Shungin, D.; Ferreira Zandoná, A.G.; Schmadeke, P.; Basta, P.V.; Luo, J.; Cantrell, J.; Pahel, T.D.; Meyer, B.D.; Divaris, K. Protocols, Methods, and Tools for Genome-Wide Association Studies (GWAS) of Dental Traits. In *Methods in Molecular Biology*; Humana Press: New York, NY, USA, 2019; Volume 1922, pp. 321–338. https://doi.org/10.1007/978-1-4939-9012-2_38.
9. Haworth S., Shungin D., van der Tas J.T., Vucic S., Medina-Gomez C., Yakimov V., Feenstra B., Shaffer J.R., Lee M.K., Standl M., et al. Consortium-Based Genome-Wide Meta-Analysis for Childhood Dental Caries Traits. *Hum. Mol. Genet.* **2018**, *27*, 3113–3127. https://doi.org/10.1093/hmg/ddy237
10. Lewis, D.D.; Shaffer, J.R.; Feingold, E.; Cooper, M.; Vanyukov, M.M.; Maher, B.S.; Slayton, R.L.; Willing, M.C.; Reis, S.E.; McNeil, D.W.; et al. Genetic Association of MMP10, MMP14, and MMP16 with Dental Caries. Int J Dent. 2017, 2017, 8465125. https://doi.org/10.1155/2017/8465125.
11. Chapple, I.L.C.; Bouchard, P.; Cagetti, M.G.; Campus, G.; Carra, M.C.; Cocco, F.; Nibali, L.; Hujoel, P.; Laine, M.L.; Lingström, P.; et al. Interaction of Lifestyle, Behaviour or Systemic Diseases with Dental Caries and Periodontal Diseases: Consensus Report of Group 2 of the Joint EFP/ORCA Workshop on the Boundaries between Caries and Periodontal Diseases. *J. Clin. Periodontol.* **2017**, *44* (Suppl. 18), S39–S51. https://doi.org/10.1111/jcpe.12685

12. Eckert, S.; Feingold, E.; Cooper, M.; Vanyukov, M.M.; Maher, B.S.; Slayton, R.L.; Willing, M.C.; Reis, S.E.; McNeil, D.W.; Crout, R.J.; et al. Variants on Chromosome 4q21 near PKD2 and SIBLINGs Are Associated with Dental Caries. J. Hum. Genet. 2017, 62, 491–496. https://doi.org/10.1038/jhg.2016.161.
13. Filho, A.V.A.; Calixto, M.S.; Deeley, K.; Santos, N.M.; Rosenblatt, A.; Vieira, A.R. MMP20 rs1784418 Protects Certain Populations against Caries. *Caries Res.* **2017**, *51*, 46–51. https://doi.org/10.1159/000450699.
14. Nibali, L.; Di Iorio, A.; Tu, Y.K.; Vieira, A.R. Host Genetics Role in the Pathogenesis of Periodontal Disease and Caries. *J. Clin. Periodontol.* **2017**, *44*, 13–21. https://doi.org/10.1111/jcpe.12639.
15. Sofer T., Shaffer J.R., Graff M., Qi Q., Stilp A.M., Gogarten S.M., North K.E., Isasi C.R., Laurie C.C., Szpiro A.A. Meta-Analysis of Genome-Wide Association Studies with Correlated Individuals: Application to the Hispanic Community Health Study/Study of Latinos (HCHS/SOL). *Genet. Epidemiol.* **2016**, *40*, 492–501. https://doi.org/10.1002/gepi.21981 .
16. Morrison, J.; Laurie, C.C.; Marazita, M.L.; Sanders, A.E.; Offenbacher, S.; Salazar, C.R.; Conomos, M.P.; Thornton, T.; Jain, D.; Laurie, C.A.; et al. Genome-Wide Association Study of Dental Caries in the Hispanic Communities Health Study/Study of Latinos (HCHS/SOL). Hum. Mol. Genet. 2016, 25, 807–816. https://doi.org/10.1093/hmg/ddv506.
17. Shaffer, J.R.; Wang, X.; Feingold, E.; Lee, M.; Begum, F.; Weeks, D.E.; Cuenco, K.T.; Barmada, M.M.; Wendell, S.; Crosslin, D.R.; et al. Effects of Enamel Matrix Genes on Dental Caries Are Moderated by Fluoride Exposures. *Hum. Genet.* **2015**, *134*, 159–167. https://doi.org/10.1007/s00439-014-1504-7
18. Stanley, B.O.; Feingold, E.; Cooper, M.E.; Vanyukov, M.M.; Vieira, A.R. Genetic Association of *MPPED2* and *ACTN2* with Dental Caries. *J. Dent. Res.* **2014**, *93*, 626–632. https://doi.org/10.1177/0022034514534688.
19. Wang, X.; Shaffer, J.R.; Weyant, R.J.; Cuenco, K.T.; DeSensi, R.S.; Crout, R.; McNeil, D.W.; Marazita, M.L. Genome-Wide Association Scan of Dental Caries in the Permanent Dentition. *BMC Oral Health* **2012**, *12*, 57. https://doi.org/10.1186/1472-6831-12-57.
20. Shuler, C.F. Inherited Risks for Susceptibility to Dental Caries. *J. Dent. Educ.* **2001**, *65*, 1038–1045.
21. Chen, X.; Zhang, Y.; Liu, H.; Wang, J. Correlation between HLA-DQB1 and HLA-DRB1 Gene Polymorphisms and Caries: A Systematic Review and Meta-Analysis. *J. Prev. Treat. Stomatol. Dis.* **2021**, *(12)*, 820–827.
22. Agrawal N*.,* Engberg E*.,* Furuholm J*.,* Dahlström E*.,* Viljakainen H. Genetic Risk Score to Predict the Likelihood of Dental Caries in Finnish Adolescents. *Lifestyle Genomics 2021;14:91–116, DOI: 10.1159/000517609*
23. Kumar, V.; Shah, H.; Jain, R; Atre, S.; Gaikwad, M. The Dessert Dimension: Genes Related to Sweet Taste Receptors, Sweet Taste Preferences, Its Association with Alcohol Consumption and Dental Caries Incidence—A Literature Synthesis and Scoping Review. Indian Journal of Forensic Medicine & Toxicology. 2021, 15. https://doi.org/10.37506/ijfmt.v15i2.14477.
24. Piekoszewska-Ziętek, P.; Turska-Szybka, A.; Olczak-Kowalczyk, D. State of Dentition among Twins Considering the Influence of Genetic and Environmental Factors: The Systematic Review of the Literature. Dent. Med. Probl. 2016, 14, 510–523. https://doi.org/10.17219/dmp/64077.
25. Yulianti, N.; Laksmiastuti, S.R.; Dwimega, A. The Role of Genetic on the Risk for Caries in Primary Teeth: A Scoping Review. *J. Int. Dent. Med. Res.* **2023**, *16*, 899–904.
