# Supplementary material for: Genetic Variants and Dental Caries Susceptibility: An Umbrella Review and Multilevel Meta-Analysis
Source: Genes (Basel). 2026 Jun 22;17(6):724. doi: 10.3390/genes17060724 (PMC13299435; doi:10.3390/genes17060724)
Supplement: Supplementary file 1 [file genes-17-00724-s001.zip › Supplementary Table S4.pdf]

Supplementary Table S4. Summary of genes, single nucleotide polymorphisms (SNPs), chromosomal locations, and functional classification

Article, Authors and Year	Category	Gene: SNP	Chromosome
Alkuhl et al., 2022 [19]	Taste genes	**TAS2R38**	7
		**TAS1R2**	1
Aruna et al., 2023 [6]	Immune response	**TRAV4:** rs1997533, rs8011979, rs7150049, rs1997532	14
		**TNF-α:** rs1800629	6
		**ALOX15:** rs2619112, rs7217186	17
		**MBL2:** rs7096206, rs1800450, rs11003125	10
		**DEFB1:** rs1800972, rs1799946, rs11362	8
		**LTF:** rs4547741, rs1126478, rs1126477, rs17078878, rs743658, rs2269436	3
		**LPO:** rs8178350, rs17762644, rs8178290, rs8178307, rs7209537, rs3744093, rs8178329, rs8178281	17
		**MASP2:** rs72550870	1
		**HLA:** rs3763305	6
		**DRB1:** rs3763305	6
Cavallari et al., 2019 [5]	Enamel formation	**ACE (I/D)**	17
		**BMP2**	20
		**SPP1**	4
		**DSPP**	4
		**TIMP2**	17
		**TIMP1**	23
		**MMP20**	11
		**MMP2**	16
		**MMP9**	20
		**MMP3**	11
		**MMP13**	11
		**AMELX**	23
		**ENAM**	4
		**AMBN**	4
		**TUFT1**	1
		**KLK4**	19
		**DLX3**	17
		**CDX2**	13
	Transporter genes	**GLUT2**	3
	Immune response	**MBL**	10
		**DEFB1**	8
		**MBL2**	10
		**MASP2**	1
		**HLA-DR4**	6
		**HLA-DQ4**	6

		**HLA-DQ5**	6
		**HLA-DQ6**	6
		**HLA-DQ2**	6
		**FCN2**	9
	Immune response	**ALOX15**	17
	RNA Processing	**TFIP11**	22
	Immune response	**LTF**	3
	Salivary gland function	**MUC7**	4
		**AQP5**	12
	Salivary defense	**MUC5B**	11
	pH regulation	**CA6**	1
	Salivary Protein	**MG1**	–
		**MG2**	–
	Salivary Protein (PRP)	**DB**	12
		**PRP1**	12
		**PR**	12
		**PS**	12
		**PMF**	12
		**PB**	12
		**PA**	12
		**PIF**	12
	Taste genes	**TAS2R38**	7
		**TAS1R2**	1
		**TAS1R3**	1
	Vitamin D Receptor	**VDR ApaI**	12
		**VDR FokI**	12
		**VDR TaqI**	12
Chisini et al., 2025 [20]	Vitamin D Receptor	**VDR:** rs731236, rs1544410, rs7975232, rs10735810, rs11568820, rs739837, rs2228570	12
Chisini et al., 2021 [18]	Taste genes	**TAS2R38:** rs713598, rs1726866, rs10246939	7
		**TAS1R2:** rs4920566, rs9701796, rs35874116, rs3935570	1
		**TAS1R3:** rs307355	1
	Transporter genes	**GLUT2:** rs5400, rs1499821, rs5398, rs11924032	3
Chisini et al., 2020b [28]	Enamel formation	**AMBN:** rs34538475, rs4694075	4
		**AMELX:** rs17878486, rs2106416, rs5933871, rs5934997, rs6639060, rs946252, rs7052450	X
		**BMP2:** rs1884302	20
		**BMP4:** rs2761887	14
		**BMP7:** rs388286	20
		**DLX3:** rs10459948, rs11656951, rs12452477, rs16948563, rs2278163, rs2303466, rs3891034	17
		**ENAM:** rs12640848, rs2609428, rs3796703, rs3796704	4
		**KLK4:** rs198968, rs198969, rs2235091, rs2242670, rs2978642, rs2978643	19

		**MMP13:** rs2252070	11
		**MMP2:** rs243847, rs243865	16
		**MMP20:** rs1711437, rs1784418	11
		**MMP3:** rs522616	11
		**MMP9:** rs17576	20
		**TIMP1:** rs4898	X
		**TIMP2:** rs7501477	17
		**TUFT1:** rs2337360, rs4970957	1
		**TFIP11:** rs5997096, rs134136, rs3790506, rs3828054, rs7526319	22
Chisini et al., 2020a [27]	Immune response	**MBL2:** rs1800450, rs7096206, rs11003125	10
		**LTF:** rs1126478, rs1126477, rs2269436, rs743658, rs4547741, rs6441989, rs2073495, rs11716497	3
		**MASP2:** rs72550870	1
		**DEFB1:** rs11362, rs1800972, rs1799946	8
		**FCN2:** rs17514136, rs3124953	9
	Salivary defense	**MUC5B:** rs2735733, rs2249073, rs2672812, rs2672785, rs2857476	11
Chisini et al., 2023 [29]	pH regulation	**CA6:** rs17032907, rs11576766, rs142460367, rs142460368, rs2274327, rs2274328, rs2274333, rs6688840, rs2274329, rs2274330, rs2781087, rs1475734, rs6692694, rs17032875, rs12067941, rs12568902, rs6577541, rs11121278, rs1202159, rs17032912, rs7545200, rs1832262, rs6691526, rs3765965, rs2274334, rs10864376, rs3737665, rs12138897, rs3765964, rs6577546, rs6680186, rs7533137, rs7513804	1
	Water transport	**AQP2:** rs467323, rs10875989	12
	Salivary gland function	**AQP5:** rs3759129, rs1996315, rs923911, rs461872	12
	Salivary defense	**MUC5B:** rs2735733, rs2249073, rs2672812, rs2672785, rs2857476	11
Hatipoğlu et al., 2019 [4]	pH regulation	**CA6:** rs2274327, rs2274328, rs2274333	1
Hatipoğlu et al., 2020 [7]	Immune response	**DEFB1:** rs11362	8
Hemati et al., 2023 [22]	Immune response	**DEFB1:** rs11362, rs1799946, rs1800972	8
		**MBL2:** rs7096206, rs11003125, rs1800450	10
Lei et al., 2021 [8]	Vitamin D Receptor	**VDR:** rs731236	
Lips et al., 2017 [11]	Salivary proline-rich protein genes	**PRH1**	12
	pH regulation	**CA6:** rs2274327, rs2274328, rs2274333	1
	Immune response	**LTF:** rs1126478, rs6441989, rs2073495, rs11716497, rs1800972	3
		**DEFB1:** rs11362, rs1800972	8
	Salivary gland function	**MUC7**	4
	Bacteriolytic factor	**LYZL2:** rs399593	10
Li et al., 2021 [1]	Enamel formation	**AMBN:** rs34538475, rs4694075, rs496502	4

		**AMELX:** rs17878486, rs2106416, rs5933871, rs5934997, rs6639060, rs946252	X
		**ENAM:** rs12640848, rs36064169, rs3796703, rs3796704, rs3806804, rs7671281	4
		**KLK4:** rs198969, rs2235091, rs2242670, rs2978642, rs2978643	19
		**MMP13:** rs2252070, rs478927, rs597315	11
		**MMP2:** rs2287074, rs243847, rs243865	16
		**MMP3:** rs522616, rs679620	11
		**MMP8:** rs17099443, rs3765620	11
		**MMP9:** rs17576	20
		**MMP20:** rs1711437, rs1784418	11
		**TFIP11:** rs134136, rs5997096	22
		**TUFT1:** rs2337360, rs3790506, rs3811411, rs4970957	1
Li et al., 2020 [21]	Immune response	**LTF:** rs1126478, rs11216477, rs6441989, rs2073495, rs11716497	3
Li et al., 2023 [23]	Enamel formation	**KLK4:** rs2235091	19
Molaei et al., 2022 [13]	Enamel formation	**MMP9:** rs17576	20
		**MMP13:** rs2252070	11
		**MMP20:** rs1784418	11
Motahari et al., 2024 [14]	Taste genes	**TAS1R2:** rs35874116, rs9701796	1
Najafi-Ghobadi et al., 2023 [15]	Enamel formation	**MMP13**: rs2252070	11
Nireeksha et al., 2021 [16]	Vitamin D receptor	**VDR:** rs7975232	12
Piekoszewska-Ziętek et al., 2017 [3]	Enamel formation	**BMP7:** rs72626594	20
	Enamel formation	**AMELX:** rs6639060, rs184371797, rs946252, rs200163085, rs2106416, rs17878486, rs5933871, rs5934997, rs178784860	X
	Tooth formation	**ENAM:** rs1264848, rs2609428, rs7671281, rs3796704, rs3790506	4
		**KLK4:** rs198968, rs2235091	19
		**TUFT1:** rs3790506, rs2337359, rs4970957, rs3796704	1
		**MMP13:** rs2252070	11
		**DSPP:** rs2615487	4
		**AMBN:** rs7439186, rs4694075, rs34538475	4
		**KRT75:** rs2232387	12
		**MMP20:** rs1784418	11
	Immune response	**ALOX15:** rs7217186	17
	Salivary gland function	**AQP5:** rs3759129, rs467323, rs1996315, rs10875989	12
	pH regulation	**CA6:** rs2274327, rs2274328, rs17032907, rs11576766, rs2274333, rs1086437, rs3765964, rs6680186, rs2274329	1
	Salivary gland function	**MUC7**	4
		**AQP5:** rs923911, rs1996315	12
	Immune response	**DEFB1**: rs11362, rs1800972, rs1799946	8

		**LTF:** rs4547741, rs1126478	3
		**MBL2:** rs7096206, rs1800450	10
		**MASP2:** rs72550870	1
	Taste genes	**TAS2R38:** rs713598, rs1726866, rs10246939, rs307355, rs35744813	7
		**TAS1R2:** rs35874116, rs9701796, rs4920566, rs3935570	1
		**TAS1R3:** rs307355	1
		**GNAT3:** rs2074674, rs6962693	7
	Transporter genes	**GLUT2:** rs1499821	3
Qin et al., 2024 [26]	Vitamin D Receptor	**VDR:** rs731236, rs739837, rs10735810, rs2228570, rs7975232, rs1544410, rs11568820	12
Sadeghi et al., 2021 [17]	Vitamin D Receptor	**VDR:** rs7975232, rs10735810, rs2228570, rs731236, rs1544410, rs739837	12
Sharifi et al., 2020 [9]	Enamel formation	**ENAM**: rs1264848, rs3796704, rs3796703	4
		**AMELX:** rs946252, rs17878486, rs6639060, rs2106416	X
	Immune response	**LTF:** rs1126478	3
Sharifi et al., 2021 [10]	pH regulation	**CA6:** rs2274328, rs2274333, rs2274327	1
	Enamel formation	**AMBN:** rs4694075, rs34538475	4
		**TUFT1**: rs4970957, rs3790506	1
Sharma et al., 2023 [2]	Enamel formation	**AMELX:** rs946252, rs17878486, rs5934997, rs2106416, rs6639060, rs104894738, rs104894733, rs104894737, rs2287074	X
		**AMBN:** rs34538475, rs3924573, rs7439186, rs4694075	4
		**ENAM:** rs12640848, rs3796703, rs3796704	4
		**TUFT1:** rs2337360, rs1045298, rs7526319, rs8934, rs4970957, rs10158855, rs2337359, rs12749	1
		**KLK4:** rs2235091, rs198968, rs198969, rs198966	19
		**MMP20:** rs1711437	11
		**MMP13:** rs2252070	11
		**MMP16:** rs17719876, rs17720688, rs16878625, rs6469206, rs1824717, rs7826929, rs10103111, rs2616487, rs10089111, rs17718917, rs1382104, rs1551893, rs2054415, rs1477907, rs16876790	8
		**MMP2-AS1:** rs243865	16
		**TIMP1:** rs243865	X
		**MMP9:** rs17576	20
Shojaei et al., 2026 [24]	Enamel formation	**ENAM:** rs12640848, rs3796703	4
		**AMELX:** rs17878486	X
	Immune response	**LTF:** rs1126478	3
Ślebioda et al., 2021 [12]	Immune response	**DEFB1:** rs1047031, rs11362, rs1800972, rs1799946	8
Zhou et al., 2025 [25]	Enamel formation	**BMP7, MIR4325, SPO11:** rs72626594	20
		**DLX3, DLX4:** rs16948495	17

	**NEDD9:** rs7738851	6
	**AJAP1:** rs3896439, rs4654438	1
	**COL5A1:** rs34201252	9
	**CNTNAP2:** rs288547958	7
	**ID4:** rs75833698	6
	**IGSF10, MIR5186, MIR548H2, AADACL2:** rs138769355	3
	**C5orf66:** rs1122171	5
	**MIR3660:** rs80177293	5
	**MIR4643:** rs73753796	6
Immune response	**LYZL2:** rs399593	10
	**ITGAL:** rs1064524	16
	**CXCR1, CXCR2:** rs1079204	2
	**SYPL-1, NAMPT:** rs190395159	7
	**KPNA4:** rs17236529	3
	**ST3GAL1:** rs76823412	8
Saliva	**PLUNC family:** rs17124372	20
pH regulation	**CA12:** rs72748935	15
	**CA9, TLN1:** rs766344453	9
	**ASIC2:** rs7503428	17
Taste genes	**TAS2R38, TAS2R3, TAS2R4, TAS2R25, OR9A4:** rs111979811	7
Undefined	**GNG4, LYST, B3GALNT2, TBCE, GGPS1, ARIB4D:** rs138642966	1
	**RHOU:** rs9793739	1
	**CTTNA2, LRRTM1:** rs112924349	2
	**ALLC:** rs1594318, rs872877	2
	**PDE11A:** rs6708025	2
	**PAPOLG, REL:** rs11686767	2
	**PDCD6IP:** rs74470773	3
	**PHYKP, RMND5B L:** rs67412107	5
	**DET1, ISG20, MFGE8:** rs140499777	15
	**CACNA1G, ABCC3, LUC7L3, WFIKKN2, ANKRD40, TOB1, SPAG9:** rs71381322	17
	**SUSD1, UGCG:** rs113021760	9
	**APTAX, NFX1:** rs17226825	9
	**PCDH15:** rs35324031	10
	**ANK3, CDK1, RHOBTB1:** rs116717469	10
	**CNIH:** rs4251631	14
	**SPTSSA:** rs75459295	14
	**BCOR:** rs17145638	X
	**ACOT9, PRDX4, SAT1, APOO:** rs141563584	X
	**ADAMTS3:** rs10805050, rs788911, rs1383934	4

References:

1. Li, X.; Liu, D.; Sun, Y.; Yang, J.; Yu, Y. Association of Genetic Variants in Enamel-Formation Genes with Dental Caries: A Meta- and Gene-Cluster Analysis. *Saudi J. Biol. Sci.* **2021**, *28*, 1645–1653.
2. Sharma, A.; Patil, S.S.; Muthu, M.S.; Venkatesan, V.; Kirubakaran, R.; Nuvvula, S.; Arockiam, S. Single Nucleotide Polymorphisms of Enamel Formation Genes and Early Childhood Caries—Systematic Review, Gene-Based, Gene Cluster and Meta-Analysis. *J. Indian Soc. Pedod. Prev. Dent.* **2023**, *41*, 3–15.
3. Piekoszewska-Ziętek, P.; Turska-Szybka, A.; Olczak-Kowalczyk, D. Single Nucleotide Polymorphism in the Aetiology of Caries: Systematic Literature Review. *Caries Res.* **2017**, *51*, 425–435.
4. Hatipoglu, O.; Saydam, F. Effects of the Carbonic Anhydrase Vi Gene Polymorphisms on Dental Caries: A Meta-Analysis. *Dent. Med. Probl.* **2019**, *56*, 395–400.
5. Cavallari, T.; Arima, L.Y.; Ferrasa, A.; Moysés, S.J.; Moysés, S.T.; Herai, R.H.; Werneck, R.I. Dental Caries: Genetic and Protein Interactions. *Arch. Oral Biol.* **2019**, *108*, 104522.
6. Aruna, P.; Patil, S.S.; Muthu, M.S.; Vettriselvi, V.; Arockiam, S.; Kirubakaran, R.; Sivakumar, N. Association between Polymorphisms of Immune Response Genes and Early Childhood Caries—Systematic Review, Gene-Based, Gene Cluster, and Meta-Analysis. *J. Genet Eng. Biotechnol.* **2023**, *21*, 124.
7. Hatipoğlu, Ö.; Saydam, F. Association between Rs11362 Polymorphism in the Beta-Defensin 1 (Defb1) Gene and Dental Caries: A Meta-Analysis. *J. Oral Biosci.* **2020**, *62*, 272–279.
8. Lei, W.; Tian, H.; Xia, Y. Association between the Taqi (Rs731236 T>C) Gene Polymorphism and Dental Caries Risk: A Meta-Analysis. *Genet Test. Mol. Biomark.* **2021**, *25*, 368–375.
9. Sharifi, R.; Jahedi, S.; Mozaffari, H.R.; Imani, M.M.; Sadeghi, M.; Golshah, A.; Moradpoor, H.; Safaei, M. Association of Ltf, Enam, and Amelx Polymorphisms with Dental Caries Susceptibility: A Meta-Analysis. *BMC Oral Health* **2020**, *20*, 132.
10. Sharifi, R.; Shayan, A.; Jamshidy, L.; Mozaffari, H.R.; Hatipoğlu, Ö.; Tadakamadla, S.K.; Sadeghi, M. A Systematic Review and Meta-Analysis of Ca Vi, Ambn, and Tuft1 Polymorphisms and Dental Caries Risk. *Meta Gene* **2021**, *28*, 100866.
11. Lips, A.; Antunes, L.S.; Antunes, L.A.; Pintor, A.V.B.; Santos, D.A.B.; Bachinski, R.; Küchler, E.C.; Alves, G.G. Salivary Protein Polymorphisms and Risk of Dental Caries: A Systematic Review. *Braz. Oral Res.* **2017**, *31*, e41.
12. Ślebioda, Z.; Woźniak, T.; Dorocka-Bobkowska, B.; Woźniewicz, M.; Kowalska, A. Beta-Defensin 1 Gene Polymorphisms in the Pathologies of the Oral Cavity-Data from Meta-Analysis: Association Only with Rs1047031 Not with Rs1800972, Rs1799946, and Rs11362. *J. Oral Pathol. Med.* **2021**, *50*, 22–31.
13. Molaei, Z.; Motahari, P. Association of Mmp9, Mmp13 and Mmp20 Genes Polymorphism with Dental Caries: A Meta-Analysis. *Pediatr. Dent. J.* **2022**, *32*, 131–140.
14. Motahari, P.; Molaei, Z.; Adhami, Z.E. Association of Tas1r2 (Rs35874116 or Rs9701796) Gene Polymorphism with Dental Caries: A Systematic Review and Meta-Analysis. *Open Dent. J.* **2024**, *18.* http://dx.doi.org/10.2174/0118742106275130240109051833
15. Najafi-Ghobadi, K.; Rajabi-Moghaddam, M.; Abbaszadeh, H. The Association between Mmp13 Rs2252070 Polymorphism and Caries Susceptibility: A Systematic Review and Meta-Analysis. *Hum. Gene* **2023**, *35*, 201143.
16. Nireeksha, M.; Hegde, N.; Suchetha Kumari, N. Antimicrobial Peptide Cathelicidin and Vitamin D Receptor Gene Polymorphism in Oral Health. *Indian J. Forensic Med. Toxicol.* **2021**, *15*, 2011–2017.
17. Sadeghi, M.; Golshah, A.; Godiny, M.; Sharifi, R.; Khavid, A.; Nikkerdar, N.; Tadakamadla, S.K. The Most Common Vitamin D Receptor Polymorphisms (Apai,Foki, Taqi, Bsmi, and Bgli) in Children with Dental Caries: A Systematic Review and Meta-Analysis. *Children* **2021**, *8*, 302.
18. Chisini, L.A.; Cademartori, M.G.; Conde, M.C.M.; Costa, F.d.S.; Salvi, L.C.; Tovo-Rodrigues, L.; Correa, M.B. Single Nucleotide Polymorphisms of Taste Genes and Caries: A Systematic Review and Meta-Analysis. *Acta Odontol. Scand.* **2021**, *79*, 147–155.
19. Alkuhl, H.; Morgan, R.; Koletsi, D.; Kavvadia, K. Genetic Taste Sensitivity and Dental Caries in Children and Adolescents: A Systematic Review and Meta-Analysis. *Int. J. Paediatr. Dent.* **2022**, *32*, 204–222.

20. Chisini, L.A.; Salvi, L.C.; de Carvalho, R.V.; Santos Costa, F.d.; Demarco, F.F.; Correa, M.B. Pathways of the Vitamin D Receptor Gene and Dental Caries: A Systematic Review and Meta-Analysis. *Arch. Oral Biol.* **2025**, *173*, 106195.
21. Li, X.; Su, Y.; Liu, D.; Yang, J. The Association between Genetic Variants in Lactotransferrin and Dental Caries: A Meta- and Gene-Based Analysis. *BMC Med. Genet* **2020**, *21*, 114.
22. Hemati, G.; Imani, M.M.; Choubsaz, P.; Inchingolo, F.; Sharifi, R.; Sadeghi, M.; Tadakamadla, S.K. Evaluation of Beta-Defensin 1 and Mannose-Binding Lectin 2 Polymorphisms in Children with Dental Caries Compared to Caries-Free Controls: A Systematic Review and Meta-Analysis. *Children* **2023**, *10*, 232
23. Li, Y.; Zhang, L.; Cen, W.; Yuan, Y. Association of Klk4 Rs2235091 Polymorphism with Susceptibility to Dental Caries: A Systematic Review and Meta-Analysis. *Front. Pediatr.* **2023**, *11*, 1236000.
24. Shojaei, D.; Sadat Mohammadipour, H.; Sekandari, S.; Dehghani, M.; Mohajertehran, F. The Role of Ltf, Enam, and Amelx Gene Polymorphisms in Dental Caries Susceptibility: A Meta-Analysis. *Curr. Genet. Med. Rep.* **2026**, *14*, 4.
25. Zhou, K.; Gao, C.; Wu, J.; Banerjee, A.; Ide, M.; Kang, J. Genome-Wide Association Studies on Dental Caries: A Systematic Review. *Caries Res.* **2025**. Available online: https://doi.org/10.1159/000548693 (accessed on 12 May 2026).
26. Qin, X.; Wang, M.; Wang, L.; Xu, Y.; Xiong, S. Association of Vitamin D Receptor Gene Polymorphisms with Caries Risk in Children: A Systematic Review and Meta-Analysis. *BMC Pediatr.* **2024**, *24*, 650.
27. Chisini, L.A.; Cademartori, M.G.; Conde, M.C.M.; Santos Costa, F.D.; Tovo-Rodrigues, L.; de Carvalho, R.V.; Demarco, F.F.; Correa, M.B. Genes and Snps in the Pathway of Immune Response and Caries Risk: A Systematic Review and Meta-Analysis. *Biofouling* **2020**, *36*, 1100–1116.
28. Chisini, L.A.; Cademartori, M.G.; Muniz Conde, M.C.; Tovo-Rodrigues, L.; Correa, M.B. Genes in the Pathway of Tooth Mineral Tissues and Dental Caries Risk: A Systematic Review and Meta-Analysis. *Clin. Oral Investig.* **2020**, *24*, 3723–3738.
29. Chisini, L.A.; de Carvalho, R.V.; Santos Costa, F.D.; Salvi, L.C.; Demarco, F.F.; Correa, M.B. Genes and Single Nucleotide Polymorphisms in the Pathway of Saliva and Dental Caries: A Systematic Review and Meta-Analysis. *Biofouling* **2023**, *39*, 8–23.
