# Supplementary material for: Genetic Variants and Dental Caries Susceptibility: An Umbrella Review and Multilevel Meta-Analysis
Source: Genes (Basel). 2026 Jun 22;17(6):724. doi: 10.3390/genes17060724 (PMC13299435; doi:10.3390/genes17060724)
Supplement: Supplementary file 1 [file genes-17-00724-s001.zip › Supplementary Table S5.pdf]

Supplementary Table S5. Detailed characteristics of included studies.

Author, year	Primary Studies SR; (MA)	Design of Primary Studies	Age (Range)	Countries of Primary Studies	Sample Size range; (total)	Independent Variables	Outcome Measures	Quality Tool	Conclusion	Funding
Shojaei et al., 2026 [1]	30; (12)	CC	2-61 yrs	TUR, BRA, KOR, POL, PHL, ARG, USA, CZE, CHN, IRN, IDN, SAU, EGY and IND	30-1354; (n=11789)	GPMs in LTF, ENAM, AMELX	DMFT/dmft and ICDAS	Q-Genie	LTF, ENAM, and AMELX polymorphism showed no consistent associations with dental caries susceptibility.	Yes
Zhou et al., 2025 [2]	16; (NM)	GWAS	2-93 yrs	USA, DNK, FIN, ESP, ARG, PHL, COL, GTM and HUN	210-53325	Genetic variants (SNPs)	DMFS/T, DFS/T,dfs/t, dmfs/t, dmftw (including WSL), DFSS, binary dental caries outcome, ICDAS, dfsPF, dfsSM, PF D1MFS, SM D1MFS	Q-Genie	GWAS findings on dental caries were inconsistent due to study heterogeneity; only NEDD9 (rs7738851) and NAMPT (rs190395159) were explored for association with dental caries in large studies.	Yes
Chisini et al., 2025 [3]	14; (13)	CC and CS	3-60 yrs	CHN, TUR, CZE, FIN, IND and BRA	60-731; (n=4944 in SR and 4425 in MA)	SNPs in VDR	DMFT, dmft, and ICDAS	JBI, GRADE, and the 10-point methodological scoring system	No individual VDR SNP showed a significant association with dental caries; However, the gene-level heterozygous model increased caries odds by 42% in East Asians.	Yes

Sharifi et al., 2021 [4]	8; (8)	CC	20 mo – 60 yrs	BRA, TUR, CHN, POL and JPN	81-355; (n=1498)	GPMs in CA6, AMBN, TUFT1	DMFT/dmft	JBI	CA6, AMBN, TUFT1 polymorphism showed no significant association with dental caries risk.	Yes
Nireeksha et al., 2021 [5]	7; (NM)	CO	NR	NR	4-400; (n=994)	Salivary levels of LL-37 and GPMs in VDR	Dental caries	General evaluation with unspecified criteria; no standard tool.	GPMs in VDR (Bsml and FokI) were associated with higher caries risk, while higher salivary LL-37 levels were associated with lower risk.	Yes (Self-funded)
Molaei et al., 2022 [6]	11; (11)	CC and CS	3-65 yrs	TUR, USA, CZE, CHN, BRA, JPN and POL	81-893; (n=4750)	GPMs in MMP9, MMP13, MMP20	DMFT/dmft	NOS	MMP13 polymorphism was associated with reduced caries risk, while MMP9 and MMP20 showed no association.	No
Najafi-Ghobadi et al., 2023 [7]	7; (7)	CC	NR	BRA, SAU, JPN, CHN, CZE and TUR	81-782; (n=2343)	GPMs in MMP13	Dental caries	JBI	No significant association was found between MMP13 (rs2252070) polymorphism and caries susceptibility.	No
Motahari et al., 2024 [8]	6; (6)	CC and CS	5-45 yrs	CHN, TUR, BRA and CZE	182-1055; (n=2978)	GPMs in TAS1R2	DMFT/dmft	NOS	TAS1R2 (rs9701796) polymorphism increased caries risk. No association was found for (rs35874116).	No
Piekoszewska-Ziętk et al., 2017 [9]	30; (NM)	NR	1-84 yrs	NR	30-12803; (n=28890)	SNPs in TDM (BMP7, AMELX, ENAM, KLK4, TUFT1, ALOX15, KRT75, MMP20, AMBN, MMP13, DSPP, ESRRB); SCF (CA6,	DMFT/DMFS and dmft/dmfs	NOS	AMELX, AQP5, and ESRRB were consistently linked to dental caries, indicating an important role in caries risk.	No

						MUC7, AQP5); IMR (DEFB1, LTF, MBL2, MASP2); and TPS (TAS2R38, TAS1R2, TAS1R3, GLUT2, GNAT3)				
Cavallari et al., 2019 [10]	51; (NM)	CC, CS and COH	2-51 yrs	USA, MEX, TUR, GTM, BRA, KOR, POL, PHL, ARG, CAN, FRA, CHN, CZE, ITA, IND, BGR and SAU	50-1819; (n=16975)	ACE I/D, ALOX15, AMBN, AMELX, APAL, AQP5, BMP2, CA6, CDX2, DB, DEFB1, DLX3, DSPP, ENAM, FOKL, FCN2, GLUT2, HLA-DR4, HLA-DQ2, HLA-DQ3, HLA-DQ4, HLA-DQ5, HLA-DQ6, KLK4, LTF, MASP2, MBL, MBL2, MG1, MG2, MMP2, MMP3, MMP9, MMP13, MMP20, MUC5B, OS, MUC7, PA, PB, PIF, PMF, PR, PRP1, SPP1, TAS1R2, TAS1R3, TAS2R38, TIMP1, TIMP2, TUFT1, TFIP11, VDR-TAQI, VDR-FOK	DMFT/dmft	NOS	A total of 27 genes (PRP1, PR, PA, MG1, MG2, AMELX, ENAM, TUFT1, KLK4, HLA- DR4, TAS1R3, TAS2R38, MBL2, MMP20, MMP2, MMP9, MMP13, GLUT2, TAS1R2, CA-VI, DEFB1, ALOX15, VDR-TAQI, MMP3, CA6, MUC5B, VDR-FOK) were associated with either increased risk or protection against dental caries.	Yes
Hatipoglu et al., 2019 [11]	4; (4)	COH and CC	6-60 yrs	BRA, CHN and TUR	178-355; (n= 932)	SNPs in CA6	DMFT/dmft	Modified NOS	No significant association was found between CA6 polymorphisms and dental caries.	No
Sharifi et al., 2020 [12]	16; (16)	CC	1.7-60 yrs	JPN, BRA, KOR, POL, FRA, TUR, CZE, CHN, IRN and SAU	71-1062; (n=5432)	GPMs in LTF, ENAM, and AMELX	DMFT/dmft	NA	Only ENAM rs3796704 was associated with increased caries risk, particularly in Caucasians.	Yes
Li et al., 2020 [13]	6; (6)	CC	3-13 yrs	BRA, USA, EUR and CHN	50-1005; (n=3389)	Genetic variants in the LTF	DMFT and radiolucency	NOS	LTF variants (rs1126478) in moderate/severe cases	Yes

									and (rs1126477), were significantly associated with dental caries.	
HatipoğluÖ et al. 2020 [14]	7; (7)	CC	2-84 yrs	USA, LVA, TUR and BRA	69-296; (n=1419)	GPMs in DEFB1	DMFT/dmft	Modified NOS	DEFB1 (rs11362) polymorphism was significantly associated with caries in permanent dentition.	No
Ślebioda et al., 2021 [15]	13; (13)	CC	NR	BRA, LVA, TUR, USA, ITA, JPN, DEU, NLD and POL	69-4224; (n=7200)	GPMs in DEFB1	Dental caries	NOS	Only (rs1047031) polymorphism showed a significant association with caries susceptibility.	NR
Li et al., 2021 [16]	21; (21)	CC	20 mo – 60 yrs	IND, KOR, POL, BRA, TUR, BGR, CHN, IRN, CZE, MEX and IDN	71-1005; (n=6589)	Genetic variants in TDM (AMELX, ENAM, MMP2, MMP20, MMP13, AMBN, TUFT1, TFIP11, KLK4, MMP9, MMP3, MMP8)	DMFT/S, dmft/s, dffs, and ICDAS	NOS	AMELX, MMP20, MMP2 and MMP13 variants were significantly associated with increased caries risk.	No
Sadeghi et al., 2021 [17]	9; (9)	CC	<18 yrs	TUR, CZE, CHN, IND and BRA	120-549; (n=2823)	GPMs in VDR (ApaI, Fokl, TaqI, BsmI and BglI)	Clinical examination of dental caries	Custom 7-criteria tool; not standardized	No association with dental caries was found for most SNPs, except FokI (rs10735810)	No
Lei et al., 2021 [18]	7; (NR)	CC	3-67 yrs	CHN, IND, TUR and CZE	120-549; (n=2470)	TaqI polymorphism in VDR	Dental caries	NOS	The C allele and CC genotype of the TaqI polymorphism were associated with increased caries risk, mainly in the permanent dentition in Asian.	No
Alkuhl et al., 2022 [19]	12; (7)	CS	Children 2-16 yrs Mean	IND, ARE, USA and TUR	38-600; (n=3400)	Genetic taste sensitivity detected by (PROP)	DMFT/S, dmft/s and WSLs	GRADE	Non-tasters of PROP showed significantly higher caries experience	No

			maternal age 18-45 yrs						than medium and super tasters.	
Hemati et al., 2023 [20]	9; (9)	CC	1-18 yrs	TUR, CHN, BRA, SAU and JPN	81-404; (n=1850)	GPMs in DEFB1 and MBL2	DMFT/dmft	Modified NOS	DEFB1 (rs11362) T allele was associated with increased caries risk in primary dentition.	No
Sharma et al., 2023 [21]	21; (12)	CC, COH and CS	20 mo – 6 yrs	TUR, POL, CHN, NOR, JPN, USA, CZE, SAU and BRA	53-1005; (n=4990)	SNPs in TDM (AMELX, ENAM, TUFT1, MMP20, AMBN, KLK4, MMP9, MMP13, MMP2, MMP3, MMP10, MMP14, MMP16, TIMP1, TIMP2, TFIP11)	ECC measured by dmft, deft and WSL	Q-Genie	AMBN, MMP9, MMP13, MMP20, KLK4 polymorphism were significantly associated with ECC.	Yes
Li et al., 2023 [22]	4; (4)	CC, COH and CS	20 mo – 34 yrs	TUR, BRA, POL and CZE	95-567; (n=1311)	GPMs in KLK4	DMFT/dmft	NOS and AHRQ	KLK4 (rs2235091) polymorphism was associated with caries susceptibility in primary, but not permanent dentition.	No
Aruna et al., 2023 [23]	15; (2)	CC, COH and CS	Children ≤6 yrs	CHN, TUR, SAU, IRN, POL, BRA, NOR and USA	37-1005; (n=5399)	SNPs and genetic variants of IMR (TRAV4, TNF-α, ALOX15, MBL2, DEFB1, LTF, LPO, HLA, MASP2)	ECC primarily measured by the dmft	Q-Genie	GPMs in TNF-α, ALOX15, TRAV4, and HLA-DRB1 were associated with ECC susceptibility; ALOX15 (rs7217186, TT) increased risk, while LTF (rs4547741, CT) was protective.	Yes
Qin et al., 2024 [24]	10; (NR)	CC and CS	3-15 yrs	CHN, TUR, CZE, IND and BRA	150-549; (n=3517)	GPMs in VDR (ApaI, BsmI, TaqI, Fokl, TaqI/BglI, Cdx-2)	DMFT/dmft and ICDAS	NOS and AXIS	FokI (rs10735810) and TaqI (rs731236) were associated with caries	Yes

									risk; TaqI was a risk factor for permanent dentition in Asian.	
Lips et al., 2017 [25]	16; (NM)	CT, CC, COH and CS	2-84 yrs	USA, BRA, POL, TUR, CHN and CZE	30-920	Salivary protein polymorphisms (DEFB1, LTF, CA6, MUC7, PRH1 and LYZL2)	DMFT/dmft and DMFS	11-point scoring sheet from a previous study	DEFB1, LTF, CA6, MUC7, LYZL2, and PRH1 polymorphism were associated with dental caries risk.	NR
Chisini et al., 2020a [26]	19; (18)	CC, COH and CS	1-84 yrs	BRA, CHN, TUR, USA, POL, LVA, CZE, ITA, SAU, IRN and JPN	69-1005; (n=6947)	SNPs in IMR (MBL2, LFT, MASP2, DEFB1, FCN2, and MUC5B)	DMFT/S, dmft/s, and ICDAS	JBI and the genetic study-specific quality assessment tool	MBL2 and MUC5B were significantly associated with increased caries risk, while DEFB1 showed variable associations depending on the SNP.	Yes
Chisini et al., 2020b [27]	25; (18)	CC, COH and CS	1-72 yrs	USA, GTM, TUR, KOR, POL, BRA, PHL, ARG, FRA, JPN, CZE, CHN and NOR	96-3600; (n=13824)	SNPs in TDM (AMBN, AMELX, BMP2, BMP4, BMP7, DLX3, ENAM, KLK4, MMP13, MMP2, MMP20, MMP3, MMP9, TFIP1, TIMP1, TIMP2, TUFT1, TFIP11)	DMFT/S, dmft/s, WSLs and ICDAS	JBI	TFIP11 and AMELX were linked to increased caries risk, whereas AMBN showed a protective effect.	Yes
Chisini et al., 2021 [28]	7; (2)	CC and COH	3-65 yrs	TUR, USA, CAN, CZE, ITA and JPN	80-2249; (n=4032)	SNPs in TPS (TAS1R2, TAS2R38, TAS1R3, GLUT2)	DMFT/S, dmft/s, and ICDAS	JBI and a modified quality scoring for genetic studies	SNPs in TPS were associated with caries; TAS2R38 (rs713598, CG) showed a protective effect.	Yes

Chisini et al., 2023 [29]	16; (10)	CC, COH and CS	3-72 yrs	TUR, BRA, USA, ARG, CHN, JPN, SWE, TUN and POL	43-1,383; (n=6207)	SNPs in SCF (CA6, AQP2, AQP5, MUC5B)	DMFT/S, dmft/s, WSLs, and ICDAS	JBI and the genetic study-specific quality assessment tool	CA6, AQP2 and AQP5 were associated with dental caries experience.	Yes

Abbreviations: Q-Genie: Quality of Genetic Association Studies tool; JBI: Joanna Briggs Institute Critical Appraisal Checklist; GRADE: Grading of Recommendations Assessment, Development and Evaluation; NOS: Newcastle–Ottawa Scale; AHRQ: Agency for Healthcare Research and Quality; CC: Case-Control Studies; GWAS: Genome-Wide Association Studies; CS: Cross-Sectional Studies; CO: Controlled Observational Studies; COH: Cohort Studies; CT: Clinical Trials; Country abbreviations are based on ISO 3166-1 alpha-3 codes; SNPs: Single nucleotide polymorphisms; GPMs: Genetic Polymorphisms; TDM: Tooth Development and Mineralization Genes; SCF: Salivary Composition and Function Genes; IMR: Immune and Inflammatory Response Genes; TPS: Taste Perception and Signaling Genes; VDR: Vitamin D Receptor Genes; ECC: Early Childhood Caries; NR: Not Reported; NA: Not Assessed; NM: No Meta-analysis

References:

1. Shojaei, Donya, Hamideh Sadat Mohammadipour, Salehe Sekandari, Mohsen Dehghani, and Farnaz Mohajertehran. "The Role of Ltf, Enam, and Amelx Gene Polymorphisms in Dental Caries Susceptibility: A Meta-Analysis." *Current Genetic Medicine Reports* 14, no. 1 (2026): 4.
2. Zhou, Ke, Chenyi Gao, Jianhua Wu, Avijit Banerjee, Mark Ide, and Jing Kang. "Genome-Wide Association Studies on Dental Caries: A Systematic Review." *Caries research* (2025).
3. Chisini, Luiz Alexandre, Luana Carla Salvi, Rodrigo Varella de Carvalho, Francine dos Santos Costa, Flávio Fernando Demarco, and Marcos Britto Correa. "Pathways of the Vitamin D Receptor Gene and Dental Caries: A Systematic Review and Meta-Analysis." *Archives of oral biology* 173 (2025): 106195.
4. Sharifi, R., A. Shayan, L. Jamshidy, H. R. Mozaffari, Ö Hatipoğlu, S. K. Tadakamadla, and M. Sadeghi. "A Systematic Review and Meta-Analysis of Ca Vi, Ambn, and Tuft1 Polymorphisms and Dental Caries Risk." *Meta Gene* 28 (2021): 100866.
5. Nireeksha, M. N. Hegde, and N. Suchetha Kumari. "Antimicrobial Peptide Cathelicidin and Vitamin D Receptor Gene Polymorphism in Oral Health." *Indian Journal of Forensic Medicine and Toxicology* 15, no. 4 (2021): 2011-17.
6. Molaei, Z., and P. Motahari. "Association of Mmp9, Mmp13 and Mmp20 Genes Polymorphism with Dental Caries: A Meta-Analysis." *Pediatric Dental Journal* 32, no. 3 (2022): 131-40.
7. Najafi-Ghobadi, K., M. Rajabi-Moghaddam, and H. Abbaszadeh. "The Association between Mmp13 Rs2252070 Polymorphism and Caries Susceptibility: A Systematic Review and Meta-Analysis." *Human Gene* 35 (2023): 201143.
8. Motahari, P., Z. Molaei, and Z. E. Adhami. "Association of Tas1r2 (Rs35874116 or Rs9701796) Gene Polymorphism with Dental Caries: A Systematic Review and Meta-Analysis." *Open Dentistry Journal* 18, no. 1 (2024).
9. Piekoszewska-Ziętek, P., A. Turska-Szybka, and D. Olczak-Kowalczyk. "Single Nucleotide Polymorphism in the Aetiology of Caries: Systematic Literature Review." *Caries Res* 51, no. 4 (2017): 425-35.
10. Cavallari, T., L. Y. Arima, A. Ferrasa, S. J. Moysés, S. Tetu Moysés, R. Hirochi Herai, and R. Iani Werneck. "Dental Caries: Genetic and Protein Interactions." *Arch Oral Biol* 108 (2019): 104522.
11. Hatipoglu, O., and F. Saydam. "Effects of the Carbonic Anhydrase Vi Gene Polymorphisms on Dental Caries: A Meta-Analysis." *Dent Med Probl* 56, no. 4 (2019): 395-400.
12. Sharifi, R., S. Jahedi, H. R. Mozaffari, M. M. Imani, M. Sadeghi, A. Golshah, H. Moradpoor, and M. Safaei. "Association of Ltf, Enam, and Amelx Polymorphisms with Dental Caries Susceptibility: A Meta-Analysis." *BMC Oral Health* 20, no. 1 (2020): 132.
13. Li, X., Y. Su, D. Liu, and J. Yang. "The Association between Genetic Variants in Lactotransferrin and Dental Caries: A Meta- and Gene-Based Analysis." *BMC Med Genet* 21, no. 1 (2020): 114.
14. Hatipoğlu, Ö, and F. Saydam. "Association between Rs11362 Polymorphism in the Beta-Defensin 1 (Defb1) Gene and Dental Caries: A Meta-Analysis." *J Oral Biosci* 62, no. 3 (2020): 272-79.
15. Ślebioda, Z., T. Woźniak, B. Dorocka-Bobkowska, M. Woźniewicz, and A. Kowalska. "Beta-Defensin 1 Gene Polymorphisms in the Pathologies of the Oral Cavity-Data from Meta-Analysis: Association Only with Rs1047031 Not with Rs1800972, Rs1799946, and Rs11362." *J Oral Pathol Med* 50, no. 1 (2021): 22-31.
16. Li, X., D. Liu, Y. Sun, J. Yang, and Y. Yu. "Association of Genetic Variants in Enamel-Formation Genes with Dental Caries: A Meta- and Gene-Cluster Analysis." *Saudi J Biol Sci* 28, no. 3 (2021): 1645-53.
17. Sadeghi, M., A. Golshah, M. Godiny, R. Sharifi, A. Khavid, N. Nikkerdar, and S. K. Tadakamadla. "The Most Common Vitamin D Receptor Polymorphisms (Apai,Foki, Taqi, Bsmi, and Bgli) in Children with Dental Caries: A Systematic Review and Meta-Analysis." *Children (Basel)* 8, no. 4 (2021).

18. Lei, W., H. Tian, and Y. Xia. "Association between the Taqi (Rs731236 T>C) Gene Polymorphism and Dental Caries Risk: A Meta-Analysis." *Genet Test Mol Biomarkers* 25, no. 5 (2021): 368-75.
19. Alkuhl, H., R. Morgan, D. Koletsi, and K. Kavvadia. "Genetic Taste Sensitivity and Dental Caries in Children and Adolescents: A Systematic Review and Meta-Analysis." *Int J Paediatr Dent* 32, no. 2 (2022): 204-22.
20. Hemati, G., M. M. Imani, P. Choubsaz, F. Inchingolo, R. Sharifi, M. Sadeghi, and S. K. Tadakamadla. "Evaluation of Beta-Defensin 1 and Mannose-Binding Lectin 2 Polymorphisms in Children with Dental Caries Compared to Caries-Free Controls: A Systematic Review and Meta-Analysis." *Children (Basel)* 10, no. 2 (2023).
21. Sharma, A., S. S. Patil, M. S. Muthu, V. Venkatesan, R. Kirubakaran, S. Nuvvula, and S. Arockiam. "Single Nucleotide Polymorphisms of Enamel Formation Genes and Early Childhood Caries - Systematic Review, Gene-Based, Gene Cluster and Meta-Analysis." *J Indian Soc Pedod Prev Dent* 41, no. 1 (2023): 3-15.
22. Li, Y., L. Zhang, W. Cen, and Y. Yuan. "Association of Klk4 Rs2235091 Polymorphism with Susceptibility to Dental Caries: A Systematic Review and Meta-Analysis." *Front Pediatr* 11 (2023): 1236000.
23. Aruna, P., S. S. Patil, M. S. Muthu, V. Vettriselvi, S. Arockiam, R. Kirubakaran, and N. Sivakumar. "Association between Polymorphisms of Immune Response Genes and Early Childhood Caries - Systematic Review, Gene-Based, Gene Cluster, and Meta-Analysis." *J Genet Eng Biotechnol* 21, no. 1 (2023): 124.
24. Qin, X., M. Wang, L. Wang, Y. Xu, and S. Xiong. "Association of Vitamin D Receptor Gene Polymorphisms with Caries Risk in Children: A Systematic Review and Meta-Analysis." *BMC Pediatr* 24, no. 1 (2024): 650.
25. Lips, Andrea, Leonardo Santos Antunes, Lívia Azeredo Antunes, Andrea Vaz Braga Pintor, Diana Amado Baptista Dos Santos, Rober Bachinski, Erika Calvano Küchler, and Gutemberg Gomes Alves. "Salivary Protein Polymorphisms and Risk of Dental Caries: A Systematic Review." *Braz Oral Res* 31 (2017): e41-e41.
26. Chisini, Luiz Alexandre, Mariana Gonzalez Cademartori, Marucs Cristian Muniz Conde, Francine Dos Santos Costa, Luciana Tovo-Rodrigues, Rodrigo Varella de Carvalho, Flávio Fernando Demarco, and Marcos Britto Correa. "Genes and Snps in the Pathway of Immune Response and Caries Risk: A Systematic Review and Meta-Analysis." *Biofouling* 36, no. 9 (2020): 1100-16.
27. Chisini, Luiz Alexandre, Mariana Gonzalez Cademartori, Marcus Cristian Muniz Conde, Luciana Tovo-Rodrigues, and Marcos Britto Correa. "Genes in the Pathway of Tooth Mineral Tissues and Dental Caries Risk: A Systematic Review and Meta-Analysis." *Clin Oral Investig* 24, no. 11 (2020): 3723-38.
28. Chisini, Luiz Alexandre, Mariana Gonzalez Cademartori, Marcus Cristian Muniz Conde, Francine Dos Santos Costa, Luana Carla Salvi, Luciana Tovo-Rodrigues, and Marcos Britto Correa. "Single Nucleotide Polymorphisms of Taste Genes and Caries: A Systematic Review and Meta-Analysis." *Acta Odontol Scand* 79, no. 2 (2021): 147-55.
29. Chisini, Luiz Alexandre, Rodrigo Varella de Carvalho, Francine Dos Santos Costa, Luana Carla Salvi, Flávio Fernando Demarco, and Marcos Britto Correa. "Genes and Single Nucleotide Polymorphisms in the Pathway of Saliva and Dental Caries: A Systematic Review and Meta-Analysis." *Biofouling* 39, no. 1 (2023): 8-23.
